# XPR1 regulates fetal liver macrophage development, identity, and pyrenocyte clearance

**DOI:** 10.1084/jem.20241587

**Published:** 2025-12-03

**Authors:** Sebastian A. Stifter, Mitchell Bijnen, Selma Tuzlak, Ekaterina Petrova, Philipp Häne, Elsa Roussel, Hannah Van Hove, Kenichi Asano, Burkhard Becher, Annika Keller, Melanie Greter

**Affiliations:** 1 Institute of Experimental Immunology, University of Zurich, Zurich, Switzerland; 2 Laboratory of Immune Regulation, School of Life Science, Tokyo University of Pharmacy and Life Sciences, Tokyo, Japan; 3 Department of Neurosurgery, Clinical Neuroscience Centre, University Hospital of Zurich, University of Zurich, Schlieren, Switzerland; 4 Neuroscience Center Zurich, University of Zurich and ETH Zurich, Zurich, Switzerland

## Abstract

Inorganic phosphate (Pi) is an essential nutrient for all organisms. It has critical functions in lipid and nucleic acid synthesis, protein signaling and bone growth. Loss-of-function mutations in Pi transporters lead to embryonic and neonatal lethality. Here, we show that the only known Pi exporter, XPR1, is critical for the development of fetal macrophages in the liver and the spleen. Single-cell RNA-seq and flow cytometry analyses in conditional mice lacking *Xpr1* in hematopoietic and/or CD206^+^ cells revealed loss of the Kupffer cell transcriptional program and a shift in the development of fetal liver monocytes towards an interferon-activated monocyte/macrophage state. Functionally, *Xpr1* deficiency in embryos led to a failure to clear nuclei expelled from erythroblasts. In adulthood, splenic red pulp and bone marrow macrophages were also reduced upon loss of intrinsic *Xpr1*. Collectively, these findings reveal that XPR1 is required for the development, identity, and function of macrophages involved in erythropoiesis.

## Teaser

Fetal liver macrophages support red blood cell production and pyrenocyte clearance in the developing embryo. Stifter et al. demonstrate that XPR1, a phosphate exporter and regulator, is required for the development and function of fetal liver macrophages.

## Graphical Abstract

**Figure:**
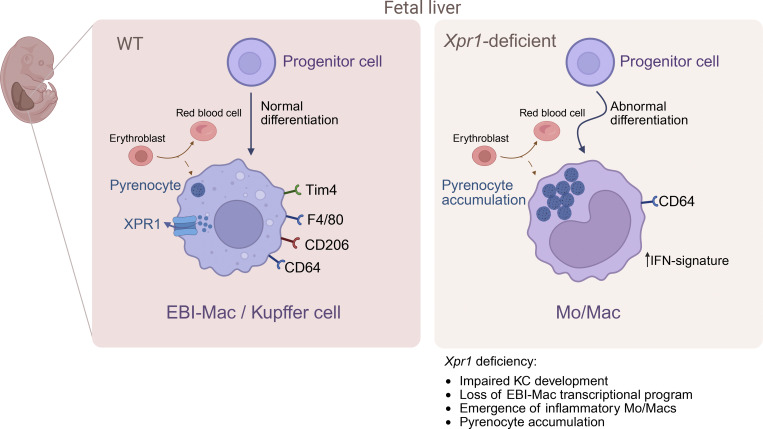

## Introduction

Inorganic phosphate (Pi) is essential for diverse functions including fetal growth, development, and metabolism, and its dyshomeostasis can lead to abnormal bone mineralization or ectopic tissue calcifications (Xu et al., 2020; Berndt and Kumar, 2007). Pi homeostasis is tightly regulated by a small number of highly conserved transporter molecules; the phosphate importer family of solute carrier (SLC) SLC20 and SLC34 proteins (Forster et al., 2013); and the only known metazoan phosphate exporter xenotropic and polytropic retrovirus receptor 1 (XPR1) (Giovannini et al., 2013). Pi transporters are critical for all life, and deletion of either *Slc20a1*, *Slc34a2,* or *Xpr1* in mice leads to growth defects and embryonic or neonatal lethality (Festing et al., 2009; Shibasaki et al., 2009; Xu et al., 2020). As the only phosphate exporter in multicellular organisms, little is known about the function(s) of XPR1. In humans, loss-of-function mutations in *XPR1* cause primary familial brain calcification, a neurodegenerative disease where calcification of blood vessels in basal ganglia is a diagnostic criterion (Legati et al., 2015). XPR1 is also highly expressed in the developing murine central nervous system (Yao et al., 2017 and https://www.genepaint.org), and heterozygous deletion of *Xpr1* (*Xpr1*^*LacZ/wt*^) leads to brain vascular calcifications and microgliosis (Maheshwari et al., 2023). Recent reports have solidified the role of XPR1 as a phosphate exporter (Yan et al., 2024; Lu et al., 2024; Zhang et al., 2025; He et al., 2025), while non-export functions of XPR1 in regulating intracellular phosphate homeostasis have also been suggested (Bondeson, 2025). Interestingly, besides its role in regulating phosphate levels, *Xpr1* in zebrafish is required for the development and differentiation of microglia and Langerhans cells (LCs) (Meireles et al., 2014). Whether XPR1 is also essential for the development of murine microglia and LCs or other macrophages remains unknown.

Virtually all organs in the body harbor macrophages, which contribute to innate immune defense by recognizing and phagocytosing invading pathogens. Moreover, tissue-resident macrophages (TRMs) perform important organ-specific homeostatic functions (Mass et al., 2023). Red pulp macrophages (RPMs) and Kupffer cells (KCs) are TRMs of the spleen and liver, respectively, where they are implicated in the recycling of iron-containing heme from red blood cells (Kovtunovych et al., 2010). Some types of macrophages also support erythropoiesis by providing developmental signals, shuttling iron-containing ferritin, and facilitating the enucleation of erythroblasts (Li et al., 2021). These populations are known as erythroblastic island macrophages (EBI-Macs) and are found in major hematopoietic organs, such as the adult bone marrow (BM) and the fetal liver (FL). The FL is the primary site of hematopoiesis during gestation from approximately embryonic day 12 (E12) to E15 (Crawford et al., 2010) and of erythropoiesis from E12.5 to E18.5 (Tada et al., 2006). At around E18, embryonic erythropoiesis moves from the liver to the spleen and persists there until early neonatal development, when it shifts to the BM (Wilding Crawford et al., 2010; Tada et al., 2006). Alterations in EBI-Mac function and/or development result in impaired erythropoiesis, leading to anemia and even embryonic lethality (reviewed in Li et al. [2021]). Although not considered EBI-Macs in the steady state, adult KCs and splenic RPMs can transform into EBI-Macs during erythropoietic stress (Sonoda and Sasaki, 2012).

In mice, the majority of TRMs develop during embryogenesis from erythromyeloid precursors in the yolk sac, and in adulthood, they self-renew locally with little to no input from the BM (Ginhoux and Guilliams, 2016; Cox et al., 2021). While most TRMs require colony-stimulating factor 1 receptor (CSF1R) signaling for their development and maintenance (Lelios et al., 2020), other factors that guide their organ-specific development and fate are starting to be discovered. A well-characterized niche is that of KCs in the liver, where expression of *Id3*, bone morphogenic proteins, and Notch signaling pathway ligands (*Dll1* and *Dll4*) have been found to be critical for their genesis and identity (Bonnardel et al., 2019).

In this study, we investigated the potential role of XPR1 in the development of TRM populations in mice. Using *Xpr1*-deficient and conditional *Xpr1* knock-out mice, we found that XPR1 was essential for the development, identity, and function of FL macrophages and RPMs, both involved in erythroblast maturation and erythrocyte production. The absence of FL macrophages led to an accumulation of interferon (IFN)-activated monocyte/macrophage-like cells in the FL that were unable to clear the expelled nuclei (pyrenocytes) of maturing erythrocytes. Together, these findings reveal a specific role for XPR1 in the development and function of EBI-Macs.

## Results

### *Xpr1*-deficient embryos lack FL macrophages

**Figure S1.**
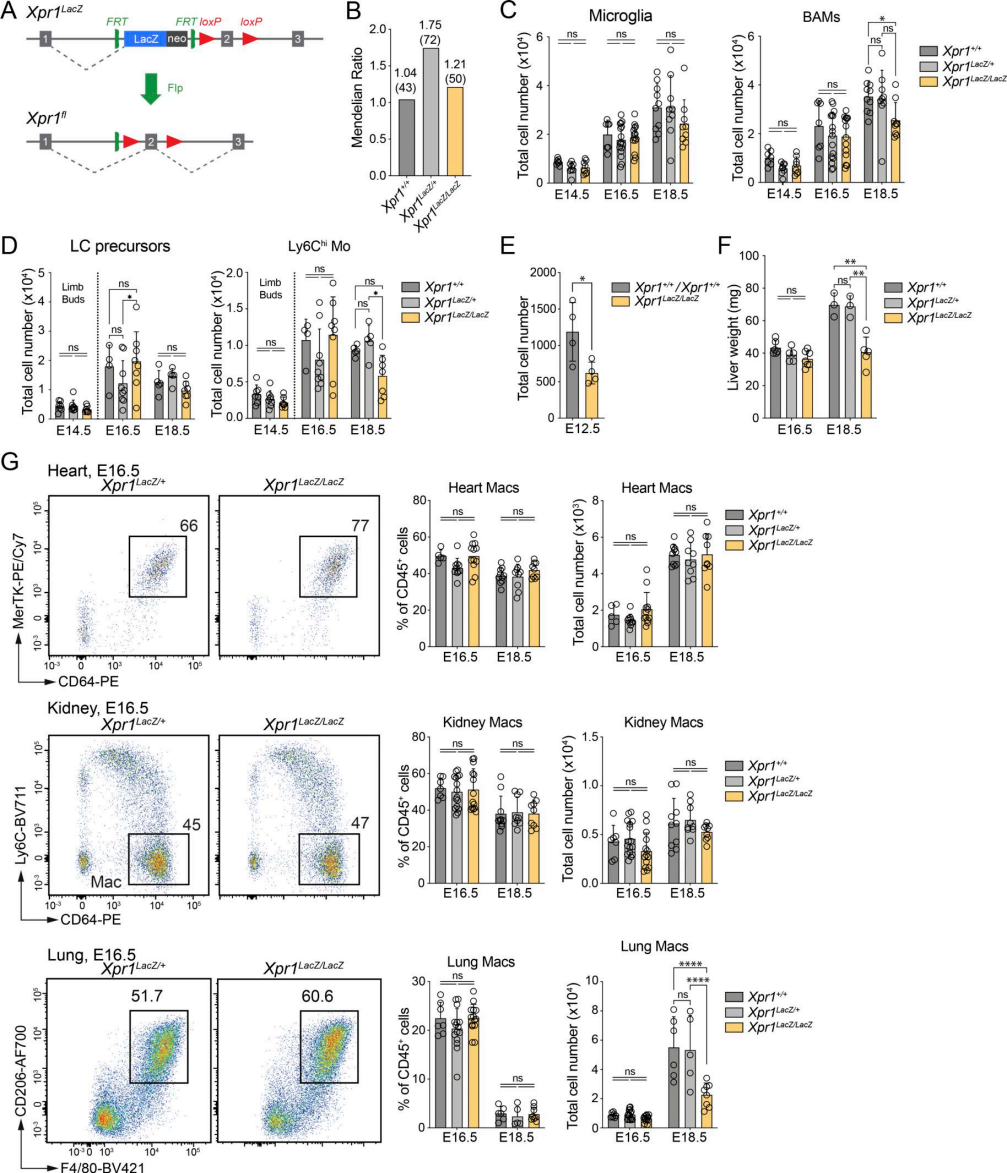
**Lung, kidney, and heart macrophages are not affected in *Xpr1*** ^***LacZ/LacZ***^ **mice. (A)** Schematic showing the *Xpr1*^*LacZ*^ allele and how crossing it to a Flp-deleter results in an *Xpr1*^*fl/fl*^ allele. **(B)** Graph showing the Mendelian distribution (and numbers) of E13.5–E18.5 embryos from an *Xpr1*^*LacZ/+*^ × *Xpr1*^*LacZ/+*^ crossing. **(C)** Graph showing the total numbers (mean ± SD) of microglia and BAMs extracted from *Xpr1*^*+/+*^, *Xpr1*^*LacZ/+*^, and *Xpr1*^*LacZ/LacZ*^ embryos at indicated time points, analyzed by flow cytometry (as in Fig. 1 A). Each time point shows data from, *n* = 7–16, pooled from at least two independent experiments. Statistical analysis was performed using one-way ANOVA with Tukey’s multiple comparisons test. **(D)** Total cell numbers (mean ± SD) of LC precursors and Ly6C^hi^ monocytes extracted from limb buds (E14.5) or skin (epidermis and dermis) of E16.5 *Xpr1*^*+/+*^, *Xpr1*^*LacZ/+*^, and *Xpr1*^*LacZ/LacZ*^ embryos, analyzed by flow cytometry. Each time point shows data from at least two independent experiments, *n* = 5–9 per group and time point. Statistical analysis was performed using one-way ANOVA with Tukey’s multiple comparisons test. Related to Fig. 1 B. **(E)** Graph showing the total numbers (mean ± SD) of FL macrophages extracted from control (*Xpr1*^*+/+*^ and *Xpr1*^*LacZ/+*^) and *Xpr1*^*LacZ/LacZ*^ embryos at E12.5, analyzed by flow cytometry. Data shown are *n* = 4 per group and are representative of two independent experiments. Statistical analysis was performed using the Student’s *t* test. Related to Fig. 1 C. **(F)** Graphs showing the weights of livers (mean ± SD) extracted from *Xpr1*^*+/+*^, *Xpr1*^*LacZ/+*^, and *Xpr1*^*LacZ/LacZ*^ embryos at indicated time points. Data shown are at least *n* = 3 per genotype and time point and are representative of at least two independent experiments. Statistical analysis was performed using the Student’s *t* test. **(G)** Representative flow cytometry plots, frequencies and total numbers (mean ± SD) of cells extracted from hearts, kidneys, and lungs of *Xpr1*^*+/+*^, *Xpr1*^*LacZ/+*^, and *Xpr1*^*LacZ/LacZ*^ embryos (pre-gated on Live CD45^+^Ly6C^−^Ly6G^−^ cells) as indicated. Each time point shows data from at least two independent experiments, *n* = 5–16 per group and time point. Statistical analysis was performed using one-way ANOVA with Tukey’s multiple comparisons test. *P < 0.05, **P < 0.01, and ****P < 0.0001. ns, not significant.

**Figure 1.**
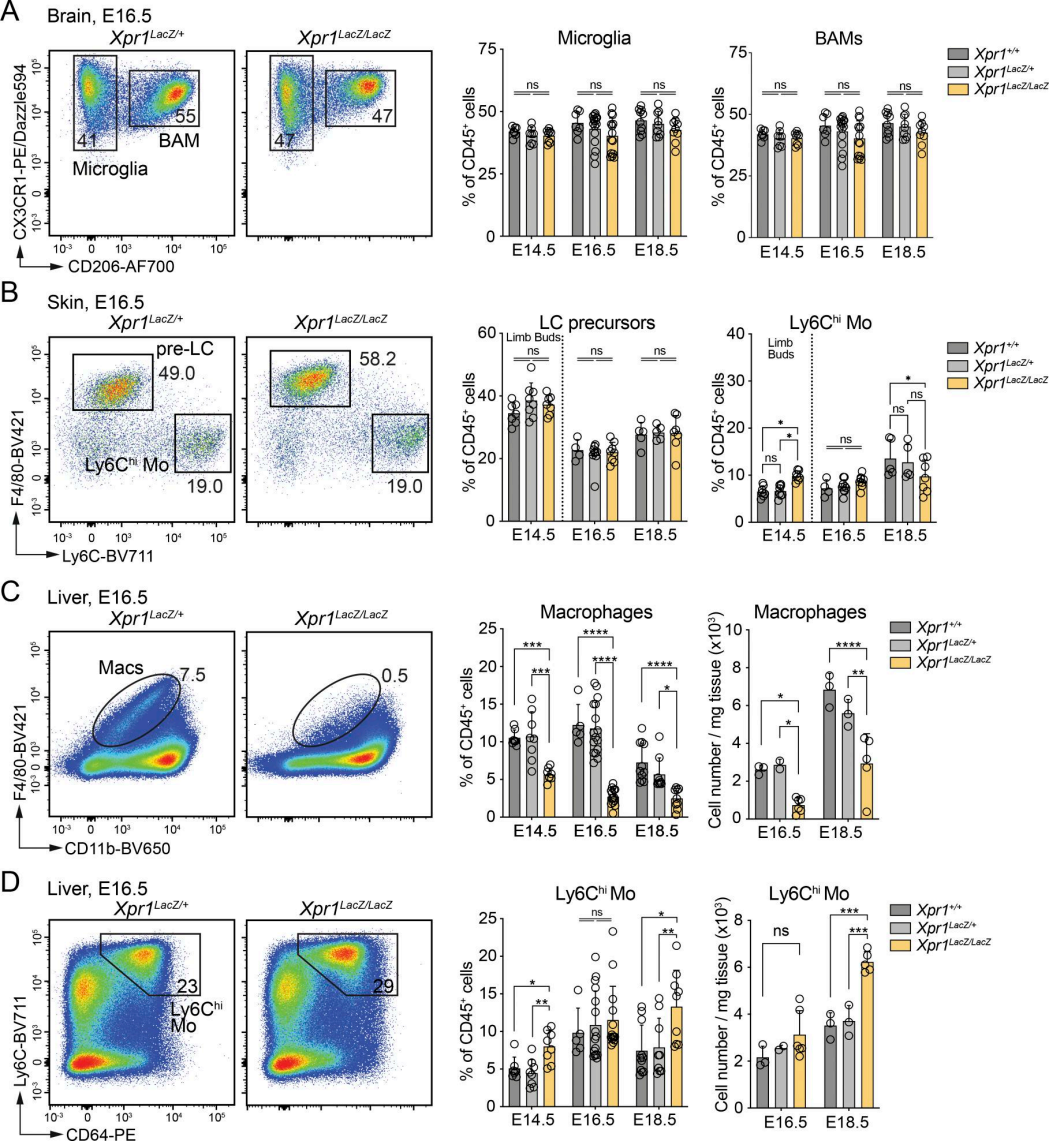
***Xpr1*** ^***Lacz/Lacz***^ **mice lack FL macrophages. (A)** Representative flow cytometry plots and frequencies (mean ± SD) of microglia (CX3CR1^+^CD206^−^) and BAMs (CX3CR1^+^CD206^+^) from brains of E16.5 *Xpr1*^*+/+*^, *Xpr1*^*LacZ/+*^, and *Xpr1*^*LacZ/LacZ*^ embryos (pre-gated on CD45^+^Ly6C^−^Ly6G^−^ cells). Each time point shows data from at least two independent experiments, *n* = 7–14 per group and time point. Statistical analysis was performed using one-way ANOVA with Tukey’s multiple comparisons test. **(B)** Representative flow cytometry plots and frequencies (mean ± SD) of LC precursors (pre-LC) and Ly6C^hi^ monocytes (Mo) extracted from limb buds (E14.5) or skin (epidermis and dermis) of E16.5 *Xpr1*^*+/+*^, *Xpr1*^*LacZ/+*^, and *Xpr1*^*LacZ/LacZ*^ embryos (pre-gated on CD45^+^Ly6G^−^CD11b^+^ cells). Each time point shows data from at least two independent experiments, *n* = 5–9 per group and time point. Statistical analysis was performed using one-way ANOVA with Tukey’s multiple comparisons test. **(C and D)** Representative flow cytometry plots, frequencies (mean ± SD) and total cell numbers (per mg tissue) (mean ± SD) of (C) macrophages (Macs) (pre-gated on CD45^+^Ly6G^−^Ly6C^−^ cells) and (D) Ly6C^hi^ monocytes (Mo) (pre-gated on CD45^+^Ly6G^−^F4/80^−^ cells) from livers of E16.5 *Xpr1*^*+/+*^, *Xpr1*^*LacZ/+*^, and *Xpr1*^*LacZ/LacZ*^ embryos. Two independent experiments for each time point, *n* = 5–16 for frequencies and *n* = 2–6 for total cell numbers. Statistical analysis was performed using one-way ANOVA with Tukey’s multiple comparisons test. *P < 0.05, **P < 0.01, ***P < 0.001, and ****P < 0.0001. ns, not significant.

To analyze whether XPR1 plays a role in the development of murine TRMs, we performed flow cytometry on cells from embryonic organs at different time points during embryogenesis. As *Xpr1*-deficient mice are not viable (Xu et al., 2020; Maheshwari et al., 2023), we used *Xpr1*^*LacZ/+*^ mice, which carry one functional *Xpr1* allele and one non-functional allele containing a *LacZ* reporter (Fig. S1 A). Crossing *Xpr1*^*LacZ/+*^ heterozygote mice resulted in no significant difference in the Mendelian ratio between *Xpr1*^*+/+*^ and *Xpr1*^*LacZ/LacZ*^ embryos, suggesting that perinatal lethality is not due to an early abort during development (Fig. S1 B). We first examined the presence of brain macrophage populations, comprising parenchymal microglia and border-associated macrophages (BAMs), which reside in the meninges, choroid plexus, and perivascular spaces (Mildenberger et al., 2022). No differences in the frequencies of microglia (CX3CR1^+^CD206^−^) or BAMs (CX3CR1^+^CD206^+^) were detected in *Xpr1*^*LacZ/LacZ*^ embryos compared to littermate controls at E14.5, E16.5, and E18.5 (Fig. 1 A and Fig. S1 C). Total cell numbers per brain were slightly reduced at E18.5 (Fig. S1 C), likely due to the smaller overall size of *Xpr1*^*LacZ/LacZ*^ embryos (Xu et al., 2020). Analyses of LCs in the skin produced similar results: we found no significant differences in the frequencies and numbers of LC (F4/80^hi^) precursors and only minor changes in Ly6C^hi^ monocytes in *Xpr1*^*LacZ/LacZ*^ embryos (Fig. 1 B and Fig. S1 D), demonstrating that the reported requirement for *Xpr1* in LCs and microglia in the zebrafish model (Meireles et al., 2014) was not conserved in mice.

Conversely, in the embryonic liver, we noted a drastic reduction in the frequency and total cell numbers of F4/80^hi^ (CD11b^lo^) FL macrophages in *Xpr1*^*LacZ/LacZ*^ embryos from E12.5 onward (Fig. S1 E and Fig. 1 C). As *Xpr1*^*LacZ/LacZ*^ embryos are smaller than wild-type (WT) and *Xpr1*^*LacZ/+*^ control littermates (Xu et al., 2020), correlating with smaller liver weights (Fig. S1 F), we calculated the total cell numbers per milligram tissue at different time points (Fig. 1 C). F4/80^hi^ FL macrophages were significantly reduced compared to controls; furthermore, we observed a concomitant increase in the frequency and numbers of FL Ly6C^hi^ monocytes during development (Fig. 1 D).

Similar to the brain and skin, we observed no significant differences in embryonic macrophages in the heart, kidney, and lung when comparing WT, *Xpr1*^*LacZ/+*^, and *Xpr1*^*LacZ/LacZ*^ embryos (Fig. S1 G). Overall, our data show that XPR1 is required for FL macrophage development but is dispensable for the embryonic development of other TRMs.

### *Xpr1* expression in hematopoietic cells is required for FL macrophage development

**Figure S2.**
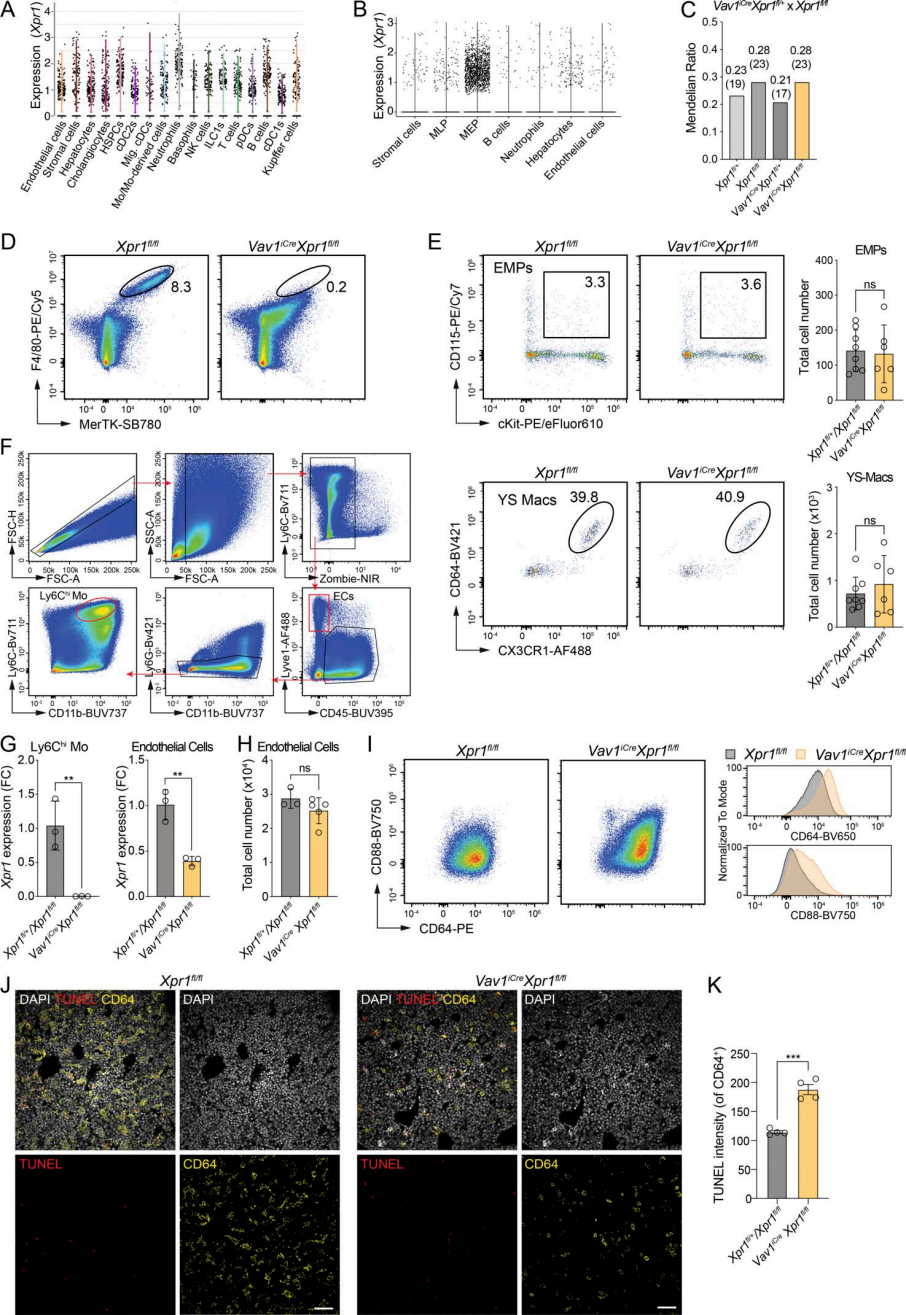
***Xpr1* expression in adult and embryonic liver cell populations. (A)** Violin plots showing the expression of *Xpr1* in indicated adult mouse liver cell populations. Data from Guilliams et al. (2022). **(B)** Violin plots showing the expression of *Xpr1* in indicated E16 embryonic liver cell populations. Data from Wang et al. (2020). **(C)** Graph showing the Mendelian distribution (and numbers) of E15.5–E18.5 embryos from a *Vav1*^*iCre*^*Xpr1*^*fl/fl*^ × *Xpr1*^*fl/fl*^ breeding. **(D)** Representative flow cytometry plots of FL macrophages from E16.5 *Xpr1*^*fl/fl*^ and *Vav1*^*iCre*^*Xpr1*^*fl/fl*^ embryos (pre-gated on CD45^+^ cells). Related to Fig. 2 A. **(E)** Representative flow cytometry plots and total cell numbers (mean ± SD) of YS EMPs and macrophages extracted from E10.5 *Xpr1*^*fl/fl*^ and *Vav1*^*iCre*^*Xpr1*^*fl/fl*^ embryos (pre-gated on CD45^+^ cells). *n* = 6–8 per group. Statistical analysis was performed using the Student’s *t* test. **(F)** Flow cytometry plots showing the gating strategy used to sort endothelial cells (CD45^−^Lyve1^+^) and Ly6C^hi^ monocytes from E16.5 *Xpr1*^*fl/fl*^ and *Vav1*^*iCre*^*Xpr1*^*fl/fl*^ FLs. **(G)** Graphs showing *Xpr1* expression in sorted endothelial cells and Ly6C^hi^ monocytes (as in F) extracted from livers of E16.5 control (*Xpr1*^*fl/fl*^ and *Xpr1*^*fl/+*^) and *Vav1*^*iCre*^*Xpr1*^*fl/fl*^ embryos. Data shown are *n* = 3 per group and are representative of two independent experiments. Statistical analysis was performed using the Student’s *t* test. **(H)** Endothelial cell numbers enumerated from E15.5 control (*Xpr1*^*fl/fl*^ and *Xpr1*^*fl/+*^) and *Vav1*^*iCre*^*Xpr1*^*fl/fl*^ embryos using flow cytometry (gated on CD31^+^CD45^−^ cells). Data shown are *n* = 3–5 per group and are representative of two independent experiments. Statistical analysis was performed using the Student’s *t* test. **(I)** Representative flow cytometry plots of FL monocytes extracted from E16.5 *Xpr1*^*fl/fl*^ and *Vav1*^*iCre*^*Xpr1*^*fl/fl*^ embryos (pre-gated on CD45^+^Lin^−^F4/80^−^CD117^−^Ly6G^−^Ly6C^+^ cells). Related to Fig. 2 F. **(J and K)** Fluorescent images of E15.5 *Xpr1*^*fl/fl*^ and *Vav1*^*iCre*^*Xpr1*^*fl/fl*^ FLs stained with DAPI, TUNEL and CD64. **(J)** Representative images, scale bars: 50 µm. **(K)** TUNEL mean staining intensity was quantified within the CD64^+^ area of E15.5 control (*Xpr1*^*fl/fl*^ and *Xpr1*^*fl/+*^) and *Vav1*^*iCre*^*Xpr1*^*fl/fl*^ FLs. Data shown are *n* = 4 per group and statistical analysis was performed using the Student’s *t* test. **P < 0.01 and ***P < 0.001. ns, not significant.

**Figure 2.**
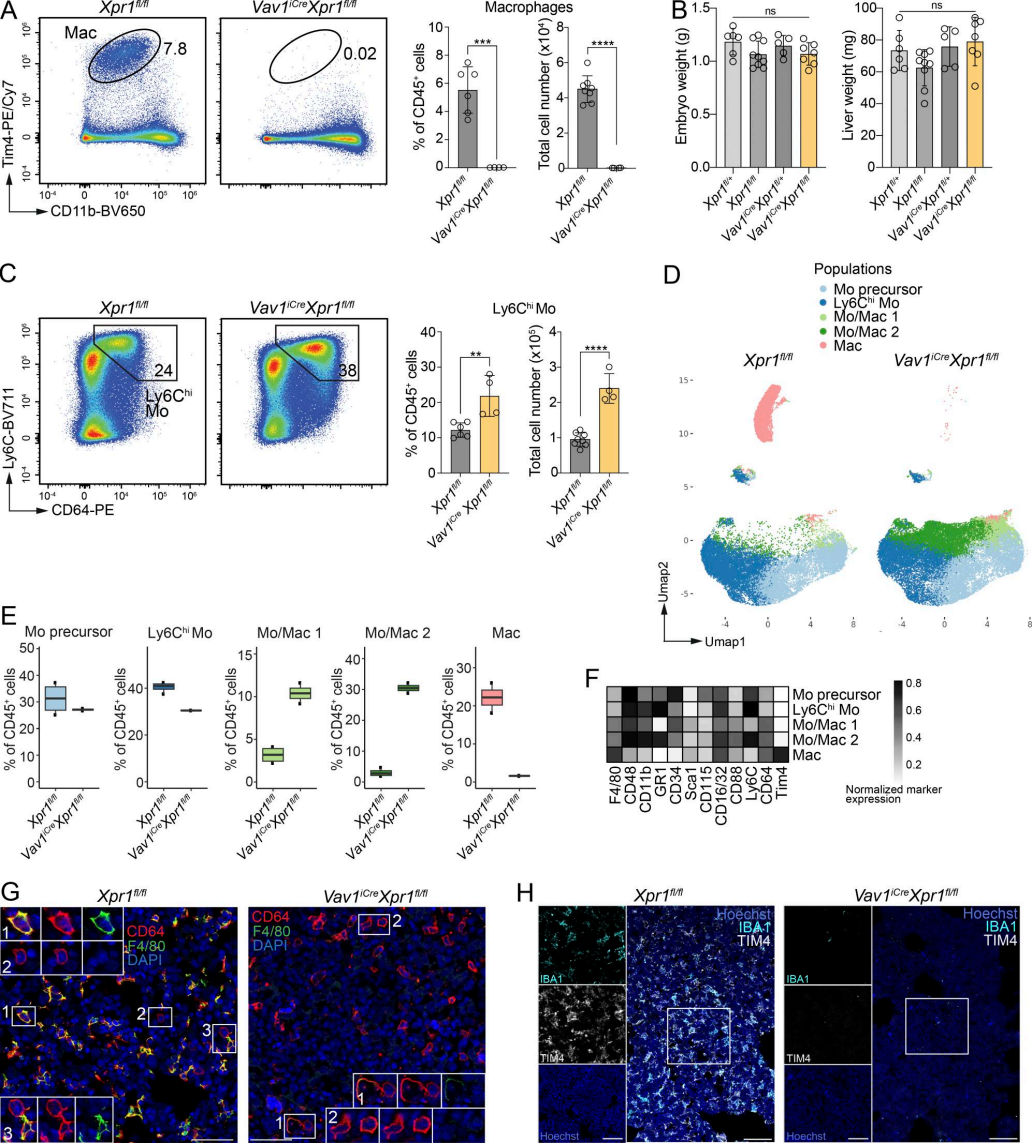
**FL macrophage development requires *Xpr1* expression in hematopoietic cells. (A)** Representative flow cytometry plots and total cell numbers (mean ± SD) of FL macrophages (Mac) extracted from E16.5 *Xpr1*^*fl/fl*^ and *Vav1*^*iCre*^*Xpr1*^*fl/fl*^ embryos (pre-gated on CD45^+^Lin^−^ [CD19^−^CD3^−^B220^−^Ter119^−^CD49b^−^CD90.2^−^] cells). Data show *n* = 4–8 per group and are pooled from two independent experiments. Statistical analysis was performed using the Student’s *t* test. **(B)** Graphs showing embryo and liver weights of E18.5 *Xpr1*^*fl/+*^, *Xpr1*^*fl/fl*^, *Vav1*^*iCre*^*Xpr1*^*fl/+*^, and *Vav1*^*iCre*^*Xpr1*^*fl/fl*^ embryos. Data show *n* = 5–9 per group and were pooled from three independent experiments. Statistical analysis was performed using one-way ANOVA with Tukey’s multiple comparison test. **(C)** Representative flow cytometry plots and total cell numbers (mean ± SD) of liver Ly6C^hi^ monocytes (Mo) extracted from E16.5 *Xpr1*^*fl/fl*^ and *Vav1*^*iCre*^*Xpr1*^*fl/fl*^ embryos (pre-gated on CD45^+^Lin^−^F4/80^−^CD117^lo-int^CD48^−^ cells). Data show *n* = 4–8 per group and are pooled from two independent experiments. Statistical analysis was performed using the Student’s *t* test. **(D)** UMAP plots of myeloid cells extracted from livers of E16.5 *Xpr1*^*fl/fl*^ and *Vav1*^*iCre*^*Xpr1*^*fl/fl*^ embryos (pre-gated on CD45^+^Lin^−^CD64^+^F4/80^+^MHCII^−^Ly6G^−^ cells) and analyzed by flow cytometry. Data were generated from *n* = 2–4 samples per group and are representative of at least four independent experiments. **(E)** Graphs showing frequencies of cell clusters defined among CD45^+^ cells (**D**). Data show *n* = 2 per group and are representative of at least four independent experiments. **(F)** Heatmap of cell surface marker expression on cell clusters defined in D. Data were transformed and percentile normalized. **(G)** Immunohistochemistry of E16.5 *Xpr1*^*fl/fl*^ and *Vav1*^*iCre*^*Xpr1*^*fl/fl*^ livers. DAPI (blue), CD64 (red), and F4/80 (green). Insets: Magnifications of the outlined regions showing expression of all markers, CD64/DAPI, and F4/80/DAPI (from left to right). Images are representative of at least five embryos per group. Scale bar: 40 µm. **(H)** Immunohistochemistry of E15.5 *Xpr1*^*fl/fl*^ and *Vav1*^*iCre*^*Xpr1*^*fl/fl*^ livers, stained for Hoechst (blue), IBA1 (cyan), and TIM4 (white). Insets: Single stainings. Images are representative of three embryos per group. Scale bar: 100 µm in overview, 50 µm in inset. **P < 0.01, ***P < 0.001, and ****P < 0.0001. ns, not significant.

Given the observed growth defects and neonatal lethality in *Xpr1*-deficient embryos, we next sought to identify the specific *Xpr1*-expressing cell type necessary for FL macrophage development. Published single-cell RNA-sequencing (scRNA-seq) datasets from adult and FL demonstrate low but ubiquitous *Xpr1* expression in all major liver cell populations (Fig. S2, A and B, data from Guilliams et al. [2022], Wang et al. [2020]). To investigate whether XPR1 is intrinsically required in hematopoietic cells, we used *Vav1*^*i*Cre^ mice, which target the whole hematopoietic compartment, as early as E8.5 (de Boer et al., 2003; Padrón-Barthe et al., 2014). Crossing *Vav1*^*iCre*^ to *Xpr1*^fl/fl^ animals produced no viable *Vav1*^*iCre*^*Xpr1*^*fl/fl*^ offspring, indicating that, as with complete *Xpr1* deficiency, deletion of *Xpr1* in hematopoietic cells and their precursors leads to lethality, while maintaining normal Mendelian frequencies during embryogenesis (Fig. S2 C). Flow cytometry analysis of *Vav1*^*iCre*^*Xpr1*^*fl/fl*^ embryos at E16.5 revealed a profound loss of FL macrophages (Fig. 2 A). This was not due to downregulation of Tim4, as we also noticed the loss of FL macrophages using F4/80 and MerTK (Fig. S2 D). Unlike *Xpr1*^*LacZ/LacZ*^ embryos, we found no differences in embryo or liver weights between control and Cre^+^ embryos even at E18.5 (Fig. 2 B), suggesting that the growth defect observed in the total knock-out was not due to lack of *Xpr1* expression in hematopoietic cells. We did not observe differences in yolk sac (YS) CSF1R^+^c-Kit^+^ erythromyeloid progenitors (EMPs) or CX3CR1^+^CD64^+^ YS macrophages at E10.5 (Fig. S2 E), suggesting that FL macrophages, but not other EMP-derived macrophages, are specifically affected in *Vav1*^*iCre*^*Xpr1*^*fl/fl*^ mice. To assess targeting efficiency, we sorted FL monocytes (Ly6C^+^CD11b^+^)—given that FL macrophages were absent—and liver endothelial cells (Lyve1^+^CD45^−^), shown to be partially targeted in *Vav1*^*Cre*^ mice (Georgiades et al., 2002), from E16.5 embryos (Fig. S2 F) and performed qRT-PCR. Sorted monocytes exhibited an almost complete loss of *Xpr1* mRNA expression, while endothelial cells exhibited an ∼60% reduction (Fig. S2 G). Notably, liver endothelial cell numbers were not different between control and *Vav1*^*iCre*^*Xpr1*^*fl/fl*^ embryos (Fig. S2 H).

Similar to *Xpr1*-deficient embryos (Fig. 1 D), frequencies and numbers of FL monocytes (Ly6C^hi^CD64^+^) were also significantly increased in E16.5 *Vav1*^*iCre*^*Xpr1*^*fl/fl*^ embryos (Fig. 2 C), suggesting that cell-intrinsic loss of *Xpr1* in hematopoietic cells was responsible for the failure of macrophage development and an increase in monocytes. To determine whether liver monocyte and macrophage cell populations changed phenotypically, we performed high-dimensional flow cytometry on FL cells extracted from *Xpr1*^*fl/fl*^ and *Vav1*^*iCre*^*Xpr1*^*fl/fl*^ embryos at E16.5. Clustering on CD64^+^F4/80^+^ cells identified five different populations (Fig. 2, D–F). As previously observed, classical FL macrophages (Tim4^hi^F4/80^hi^) were absent in *Vav1*^*iCre*^*Xpr1*^*fl/fl*^ animals (Fig. 2, D and E). This analysis further revealed that the increase in CD11b^+^CD64^+^Ly6C^hi^ monocytes in Cre^+^ embryos was not due to an increase in Ly6C^hi^ FL monocytes themselves, but rather to the emergence of two alternate monocyte/macrophage-like (Mo/Mac) populations that were largely absent in control embryos (Fig. 2, D and E, labeled as Mo/Mac 1 and 2). These Mo/Macs expressed high levels of CD11b, CD64, CD34, and CD48 and intermediate levels of F4/80, Ly6C, and CD88 (Fig. 2 F and Fig. S2 I). Notably, they did not express the canonical FL macrophage/KC marker Tim4. We next analyzed FLs by immunofluorescence microscopy and found that, while *Xpr1*^*fl/fl*^ control livers contained a dense network of FL macrophages stained for CD64 and F4/80 (Fig. 2 G, left, insert 1 and 3) or IBA1 and TIM4 (Fig. 2 H), *Vav1*^*iCre*^*Xpr1*^*fl/fl*^ livers were almost completely devoid of F4/80^+^ and IBA1^+^TIM4^+^ cells (Fig. 2, G and H). In addition, Cre^+^ livers instead contained large numbers of spherical cells expressing high levels of CD64, reminiscent of monocytes (Fig. 2 G, right, insert 1). Moreover, *Vav1*^*iCre*^*Xpr1*^*fl/fl*^ FLs exhibited a marked increase in TUNEL signal in CD64^+^ cells, indicating elevated apoptosis in monocytes/macrophages lacking *Xpr1* (Fig. S2, J and K). These data indicate that classical FL macrophages are largely absent in *Vav1*^*iCre*^*Xpr1*^*fl/fl*^ livers, which instead generate Mo/Macs that fail to acquire a characteristic liver macrophage morphology and phenotype.

### XPR1 controls the FL macrophage developmental program

**Figure 3.**
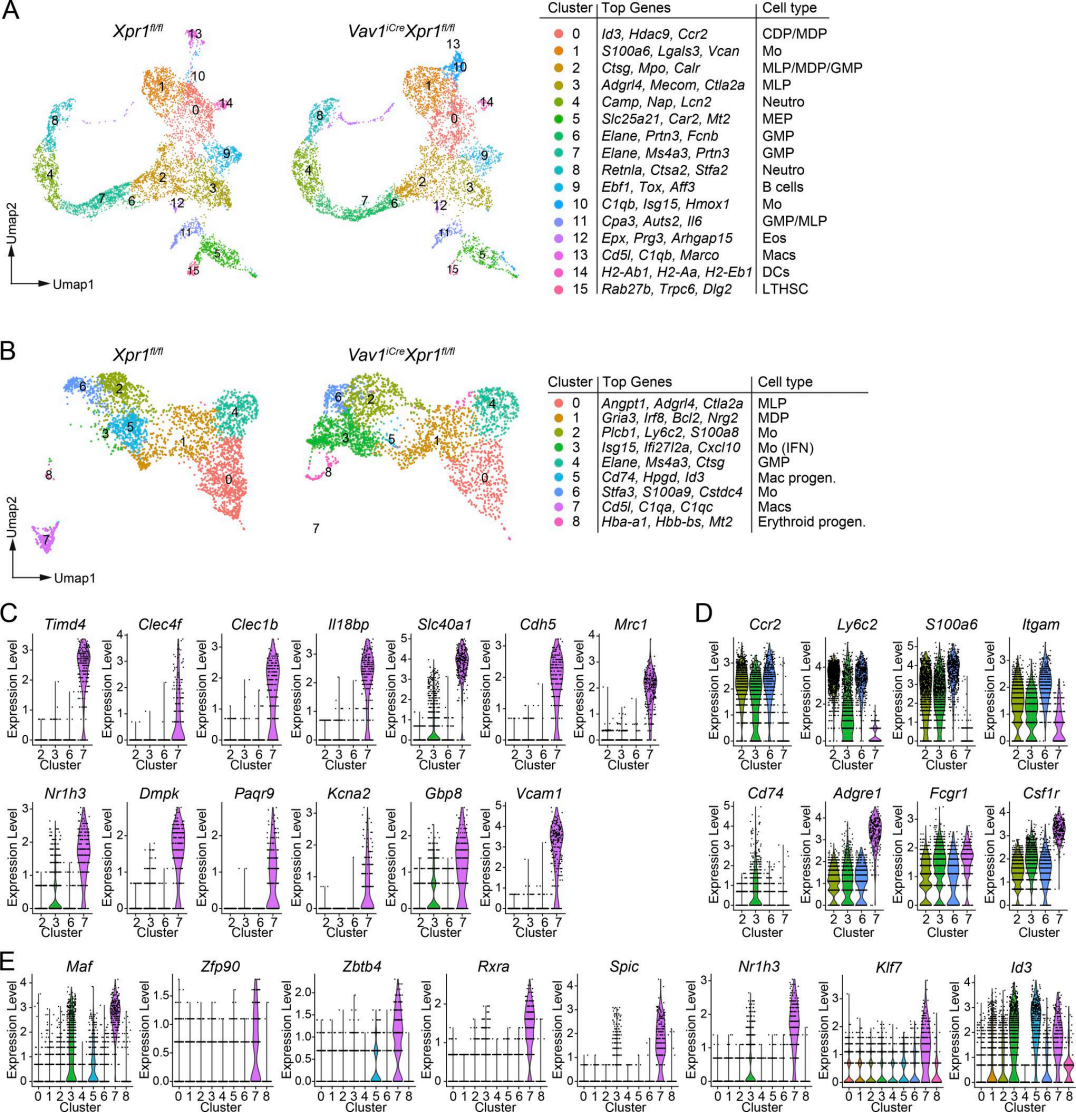
**XPR1 is required for the induction of the KC transcriptional program. (A)** UMAP plot and annotation of clusters of CD45^+^Lin^−^ (CD19^−^CD3^−^B220^−^Ter119^−^CD49b^−^CD90.2^−^) cells extracted from E15.5 *Xpr1*^*fl/fl*^ and *Vav1*^*iCre*^*Xpr1*^*fl/fl*^ livers, analyzed by scRNA-seq. Data were generated from *n* = 3 samples per genotype. **(B)** UMAP plots, guided clustering, and manual cell annotation of subsetted data from the scRNA-seq for clusters 0, 1, 2, 3, 10, and 13 in A. **(C)** Violin plots showing liver macrophage gene expression signature (Bonnardel et al., 2019) among clusters 2, 3, 6, and 7 of the scRNA-seq data in B. **(D)** Violin plots showing monocyte gene expression signature (Bonnardel et al., 2019) among clusters 2, 3, 6, and 7 of the scRNA-seq data in B. **(E)** Violin plots showing the expression of liver macrophage–associated transcription factors among clusters 0–8 of the scRNA-seq data in B.

**Figure S3.**
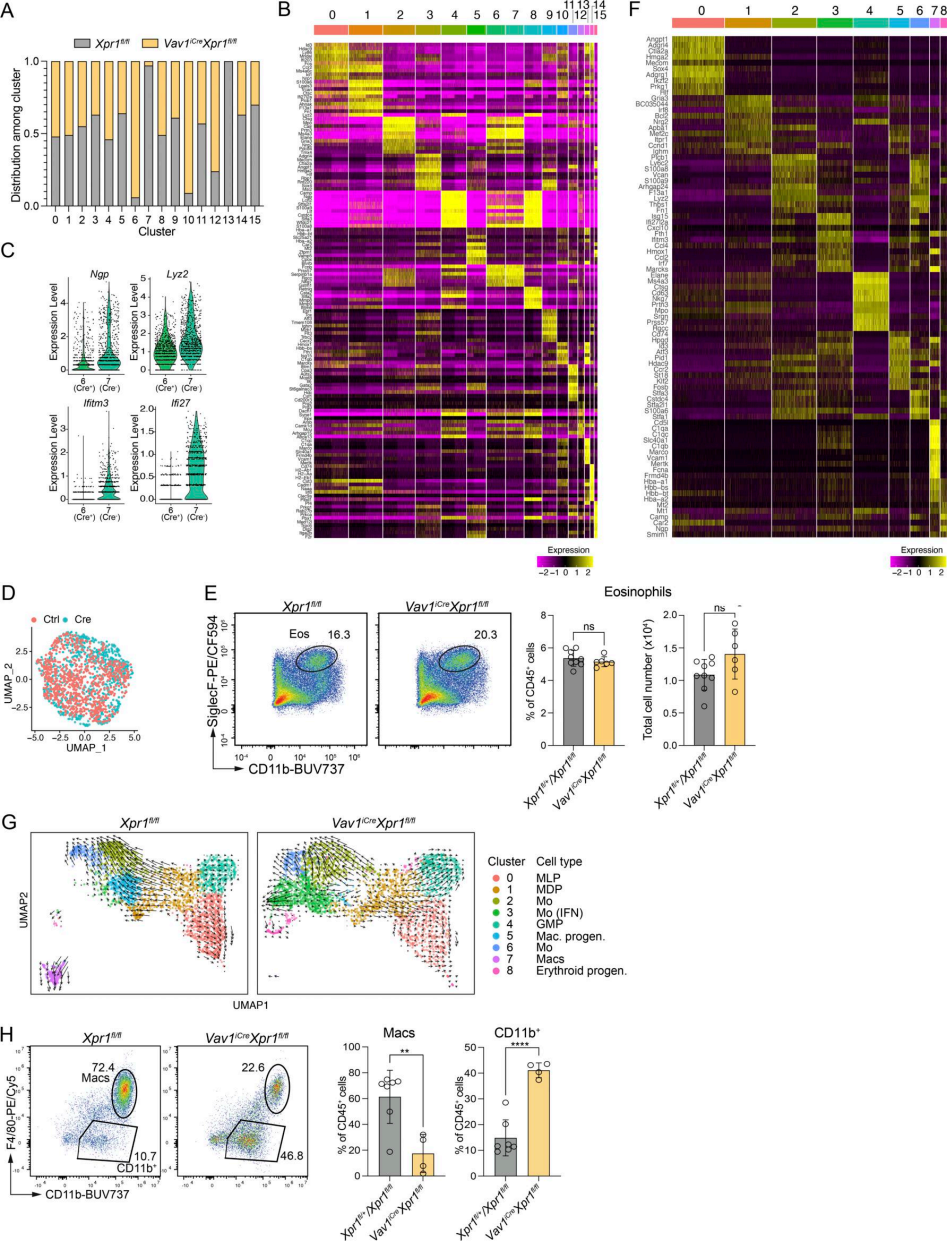
**Gene expression in CD45** ^**+**^ **FL cells. (A–D and F)** scRNA-seq of CD45^+^Lin^−^ (CD19^−^CD3^−^B220^−^Ter119^−^CD49b^−^CD90.2^−^) *Vav1*^*iCre*^*Xpr1*^*fl/fl*^ and *Xpr1*^*fl/fl*^ FL cells at E15.5, as in Fig. 3 A. **(A)** Plot showing average distribution of cell frequencies among clusters. **(B)** Heatmaps showing the top 10 DEGs of each cluster. Clusters are from Fig. 3 A. **(C)** Violin plots showing gene expression among clusters 6 (*Xpr1*^*fl/fl*^ (Cre^−^)) and 7 (*Vav1*^*iCre*^*Xpr1*^*fl/fl*^ (Cre^+^)). **(D)** UMAP plots of subsetted data (clusters 6 and 7 were used for subsetting). **(E)** Representative flow cytometry plots and frequencies and cell numbers (mean ± SD) of eosinophils (Eos) (pre-gated on CD45^+^CX3CR1^−^Ly6C^−^) extracted from E15.5 control (*Xpr1*^*fl/fl*^ and *Xpr1*^*fl/+*^) and *Vav1*^*iCre*^*Xpr1*^*fl/fl*^ FLs. Data show *n* = 6–9 per group and are pooled from two independent experiments. Statistical analysis was performed using the Student’s *t* test. **(F)** Heatmap showing the top 10 DEGs of each cluster. Clusters used are from Fig. 3 B. **(G)** RNA velocity of Fig. 3 B. Arrows indicate direction of developmental trajectory. **(H)** Representative flow cytometry plots and frequencies of macrophages (Macs) and CD11b^+^ cells (CD11b^+^) (of CD45^+^ cells) generated from E15.5 control (*Xpr1*^*fl/fl*^ and *Xpr1*^*fl/+*^) and *Vav1*^*iCre*^*Xpr1*^*fl/fl*^ FL cells cultured for 7 days *in vitro* with CSF-1. Data show *n* = 4–7 per group and are pooled from two independent experiments. Statistical analysis was performed using the Student’s *t* test. **P < 0.01 and ****P < 0.0001. ns, not significant.

The almost complete loss of canonical FL macrophages in *Vav1*^*iCre*^*Xpr1*^*fl/fl*^ embryos prompted us to investigate how XPR1 affects macrophage development. To address this question, we performed scRNA-seq on CD45^+^CD3^−^CD19^−^NK1.1^−^ cells extracted from E15.5 *Xpr1*^*fl/fl*^ and *Vav1*^*iCre*^*Xpr1*^*fl/fl*^ livers, revealing 16 cell clusters (Fig. 3 A). Cell frequencies were largely even between control and Cre^+^ samples except for clusters 6, 7, 10, 12, and 13 (Fig. 3 A and Fig. S3 A). Clusters 6 and 7 expressed *Elane*, *Ms4a3*, and *Prtn3* and were both classified as granulocyte-monocyte progenitors (GMPs) (Fig. 3 A and Fig. S3 B). Upon closer inspection, the offset UMAP coordinates of clusters 6 and 7 appeared to be due to slightly increased expression of *Ngp*, *Lyz2*, *Ifitm3*, and *Ifi27* in *Xpr1*^*fl/fl*^ (cluster 7) versus *Vav1*^*iCre*^*Xpr1*^*fl/fl*^ (cluster 6) (Fig. S3 C). Subsetting and reanalysis of these two clusters confirmed that the two populations were otherwise homogenous, however (Fig. S3 D). Cluster 12 expressed *Epx* and *Prg3* and was classified as eosinophils (Fig. 3 A and Fig. S3 B). Although this cluster appeared to be over-represented in the *Vav1*^*iCre*^*Xpr1*^*fl/fl*^ sample, flow cytometry did not show a difference in eosinophils between control and Cre^+^ conditions (Fig. S3 E).

Cluster 13 was found exclusively in *Xpr1*^*fl/fl*^, whereas cluster 10 was highly enriched in *Vav1*^*iCre*^*Xpr1*^*fl/fl*^ (Fig. 3 A and Fig. S3 A). Cluster annotation revealed these to be macrophages and monocytes, respectively (Fig. 3 A). To better understand how the loss of *Xpr1* affects macrophage development, we further subsetted monocytes (clusters 1 and 10), macrophages (cluster 13), and the precursor populations in clusters 2 (MLP/MDP/GMPs), 3 (MLPs), and 0 (CDP/MDPs). This reanalysis revealed nine Seurat clusters, including a monocyte cluster (cluster 3) present in *Vav1*^*iCre*^*Xpr1*^*fl/fl*^ livers that was virtually absent in control livers (Fig. 3 B and Fig. S3 F). This cluster was enriched with IFN-regulated genes. We also noted the loss of cluster 5 in Cre^+^ compared to control samples; this cluster expressed high levels of *Cd74*, *Hpgd*, *Id3*, *Ccr2*, and *Klf2* and is likely an FL macrophage precursor population (Fig. 3 B and Fig. S3 F). Lastly, we annotated cluster 8 (erythroid progenitors), which was rich in hemoglobin genes and genes involved in heme and iron catabolism/transport and was strongly increased in Cre^+^ livers. Comparing the monocyte and macrophage clusters using the E15 KC core gene list published by Bonnardel et al. (2019), we found that only *bona fide* FL macrophages/KCs (cluster 7) in control mice expressed high levels of *Timd4*, *Clec4f*, *Cdh5*, *Mrc1*, and *Nr1h3* (Fig. 3 C). In contrast, monocytic cells from clusters 2, 3, and 6 expressed high levels of monocyte/macrophage-related genes like *Ccr2*, *Ly6c2*, *S1006*, and *Itgam* (Fig. 3 D). While previous studies demonstrated heterogeneity within FL macrophages (Mukherjee et al., 2021; Kayvanjoo et al., 2024), we found that all FL macrophages were absent in *Vav1*^*iCre*^*Xpr1*^*fl/fl*^ mice. Overall, these data indicated that loss of *Xpr1* in *Vav1*^*iCre*^-expressing cells resulted in dysregulated FL macrophage development.

We further compared our data to published KC/FL macrophage–associated transcription factors (Bonnardel et al., 2019) and found that core transcription factors such as *Rxra* and *Zbtb4* were only expressed in fetal macrophages (cluster 7) in *Xpr1*^fl/fl^ mice. However, even though IFN-responsive monocytes (cluster 3) exhibited some expression of the liver macrophage transcription factors *Maf*, *Spic*, *Nr1h3*, and *Id3*, they failed to develop into true FL macrophages (Fig. 3 E).

To further investigate the hypothesis that loss of XPR1 affects macrophage development, we performed RNA trajectory inference using RNA velocity (Bergen et al., 2020). Compared to *Xpr1*^*fl/fl*^ control livers, the trajectory of cells in *Vav1*^*iCre*^*Xpr1*^*fl/fl*^ livers was directed towards the IFN-signature monocytes (cluster 3), suggesting an alternative developmental pathway compared to normal liver monocytes (clusters 2 and 6) or macrophages (cluster 7) (Fig. S3 G).

Lastly, we isolated FL cells from E14.5 control (*Xpr1*^*fl/+*^ and *Xpr1*^*fl/fl*^) and *Vav1*^*iCre*^*Xpr1*^*fl/fl*^ embryos and cultured them with colony-stimulating factor 1 (CSF-1) for 7 days to generate FL-derived macrophages (FLDMs). This showed that the generation of *Vav1*^*iCre*^*Xpr1*^*fl/fl*^ FLDMs was impaired compared to control livers (Fig. S3 H). Moreover, an F4/80^−^CD11b^+^ population was more pronounced in the *Vav1*^*iCre*^*Xpr1*^*fl/fl*^ sample, consistent with our *in vivo* findings of altered macrophage development (Fig. S3 H).

Altogether, these results indicate that the transcriptional program leading to the generation of FL macrophages was dysregulated in the absence of *Xpr1* and, instead, an alternative monocyte/macrophage-like population developed.

### Loss of XPR1 in FL macrophages does not alter erythrocyte enucleation

**Figure 4.**
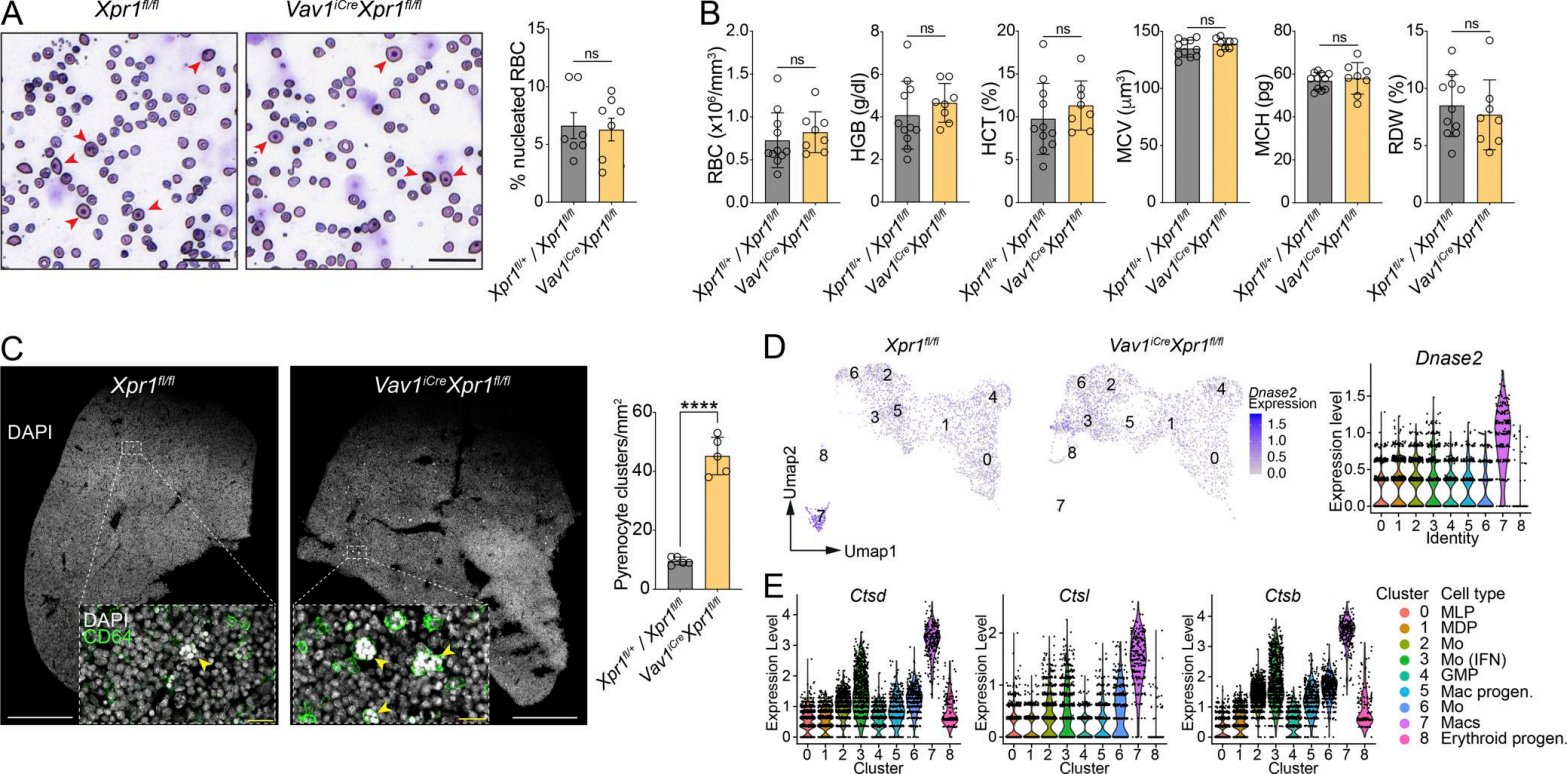
**Pyrenocyte clearance is impaired in *Vav1*** ^***iCre***^ ***Xpr1*** ^***fl/fl***^ **FLs. (A)** Microscopic images of peripheral blood smears and quantification of RBC enucleation (mean ± SEM) from E15.5 *Vav1^i^*^*Cre*^*Xpr1*^*fl/fl*^ and control littermate (*Xpr1*^*fl/fl*^ and *Xpr1*^*fl/+*^) embryos stained with modified Giemsa stain. Scale bars are 40 µm. Red arrows point to nucleated RBCs. *n* = 7–8 per genotype pooled from two independent experiments. Statistical analysis was performed using the Student’s *t* test. **(B)** Analysis of peripheral blood parameters from *Vav1*^*iCre*^*Xpr1*^*fl/fl*^ and control littermates (*Xpr1*^*fl/fl*^ and *Xpr1*^*fl/+*^) using a scil ABC plus blood counter (mean ± SD). Data shown are *n* = 8–11 pooled from three independent experiments. Statistical analysis was performed using the Student’s *t* test. **(C)** Immunohistochemistry of E15.5 *Vav1*^*iCre*^*Xpr1*^*fl/fl*^ and control littermate (*Xpr1*^*fl/fl*^ and *Xpr1*^*fl/+*^) livers and corresponding quantification of pyrenocyte clusters. The overview image shows DAPI (white) and the insets show magnification of the outlined regions and expression of DAPI (white) and CD64 (green). Yellow arrows point to CD64^+^ cells containing pyrenocytes. *n* = 5 per genotype, pooled from three independent experiments. Scale bars are 500 µm (main images) and 20 µm (insets). Statistical analysis was performed using the Student’s *t* test. **(D)** UMAP plots and violin plots of scRNA-seq data (Fig. 3 B) show the expression of *Dnase2a* among the eight cell clusters. **(E)** Violin plots of scRNA-seq data (Fig. 3 B) showing expression of the cathepsin genes *Ctsd*, *Ctsl*, and *Ctsb* among the eight cell clusters. ****P < 0.0001. ns, not significant; RBC, red blood cells; HGB, hemoglobin; HCT, hematocrit; MCV, mean corpuscular volume; MCH, mean corpuscular hemoglobin; RDW, red cell distribution width.

**Figure S4.**
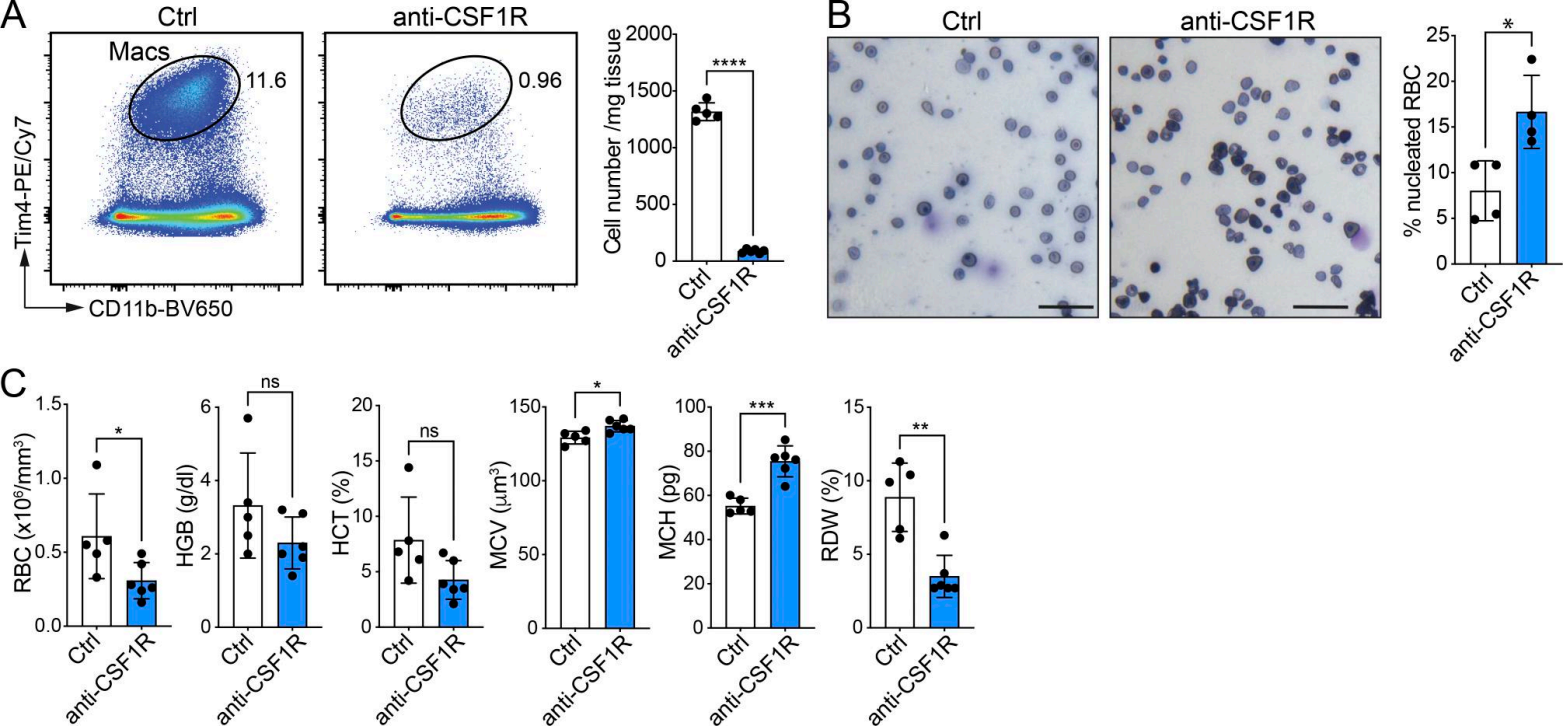
**Macrophage depletion and erythrocyte enucleation in anti-CSF1R antibody-treated embryos. (A)** Representative flow cytometry plots and total cell numbers (per mg tissue) (mean ± SD) of macrophages (pre-gated on CD45^+^Ly6G^−^ cells) from livers of anti-CSF1R antibody-treated E16.5 embryos. Data show *n* = 5–6 samples per group and are representative of two independent experiments. Statistical analysis was performed using the Student’s *t* test. **(B)** Microscopic images of peripheral blood smears and quantification of nucleated RBC (%) (mean ± SD) from untreated (Ctrl) and anti-CSF1R antibody-treated E15.5 embryos stained with Giemsa. Scale bar is 40 µm. Data show *n* = 4 samples per group and are representative of two independent experiments. Statistical analysis was performed using the Student’s *t* test. **(C)** Analysis of peripheral blood parameters from untreated (Ctrl) and anti-CSF1R antibody-treated E15.5 embryos using a blood counter. Data shown are *n* = 5–6 samples per group and are representative of two independent experiments. Statistical analysis was performed using the Student’s *t* test. *P < 0.05, **P < 0.01, ***P < 0.001, and ****P < 0.0001. ns, not significant; RBC, red blood cells; HGB, hemoglobin; HCT, hematocrit; MCV, mean corpuscular volume; MCH, mean corpuscular hemoglobin; RDW, red cell distribution width.

To investigate whether loss of FL macrophages in *Vav1*^*iCre*^*Xpr1*^*fl/fl*^ embryos leads to altered erythropoiesis, we analyzed blood smears from E15.5 embryos. No difference in the frequency of nucleated erythrocytes was found between Cre^+^ and Cre^−^ embryos (Fig. 4 A). Equally, the number of red blood cells (RBCs), hemoglobin, hematocrit, blood cell volume, or hemoglobin per RBC were also unaltered between control and *Vav1*^*iCre*^*Xpr1*^*fl/fl*^ mice at E15.5 (Fig. 4 B). As a comparison, we depleted embryonic macrophages in WT embryos by administration of anti-CSF1R antibody to pregnant dams at E8.5 (Fig. S4 A). This direct depletion of embryonic macrophages led to increased nucleated RBCs and altered blood parameters, including lower RBC count, hemoglobin, and red cell distribution width (Fig. S4, B and C). These findings suggest that although XPR1 is involved in macrophage development, the loss of canonical FL macrophages in *Vav1*^*iCre*^*Xpr1*^*fl/fl*^ embryos does not affect the enucleation of erythroblasts.

### Pyrenocytes accumulate in *Vav1*^*iCre*^*Xpr1*^*fl/fl*^ livers

While no difference in enucleation of RBCs was found, we next assessed whether the expelled nuclei (known as pyrenocytes) could be phagocytosed and cleared by macrophages in *Vav1*^*iCre*^*Xpr1*^*fl/fl*^ versus *Xpr1*^*fl/fl*^ embryos. Pyrenocyte nuclei are denser than normal nuclei due to nuclear condensation prior to expulsion, making them easily distinguishable by histology (Moras et al., 2017). E16.5 *Vav1*^*iCre*^*Xpr1*^*fl/fl*^ livers stained for CD64 and DAPI demonstrated large, dense DAPI^+^ nuclei clusters throughout the tissue (Fig. 4 C). Closer inspection of these clusters revealed nuclei engulfed by CD64^+^ cells (Fig. 4 C, insert), some of which contained upwards of 16 individual nuclei. Although we identified some nuclei-containing CD64^+^ cells in *Xpr1*^*fl/fl*^ control livers, these were less numerous and contained fewer nuclei (Fig. 4 C).

Expelled pyrenocytes are digested by cathepsins and DNase II in the lysosomes of EBI-Macs (Kawane et al., 2001). Importantly, DNase II-deficient FL macrophages do not clear pyrenocyte DNA, leading to STING-dependent type I IFN production (Ahn et al., 2012). Given our observation that Mo/Macs in *Vav1*^*iCre*^*Xpr1*^*fl/fl*^ livers have a type I IFN signature (Fig. 3 B and Fig. S3 F), we wondered if the accumulation of nuclei in these cells was due to an inability of the non-canonical Mo/Macs to digest pyrenocyte DNA. Investigating our scRNA-seq data, we found that, in contrast to *Xpr1*^*fl/fl*^ FL macrophages (cluster 7), *Vav1*^*iCre*^*Xpr1*^*fl/fl*^ IFN-activated Mo/Macs (cluster 3) expressed lower levels of *Dnase2a* and were comparable to levels found in other non-macrophage populations (Fig. 4 D). These non-canonical *Vav1*^*iCre*^*Xpr1*^*fl/fl*^ FL macrophages also expressed cathepsins at lower levels than *Xpr1*^*fl/fl*^ macrophages (Fig. 4 E). Altogether, these data indicate that XPR1 is required for the development of *bona fide* FL erythroblastic macrophages and, in their absence, pyrenocytes are not cleared by other macrophage/monocyte populations, leading to type I IFN signaling.

### Transcriptional programming and function of FL macrophages require XPR1

**Figure S5.**
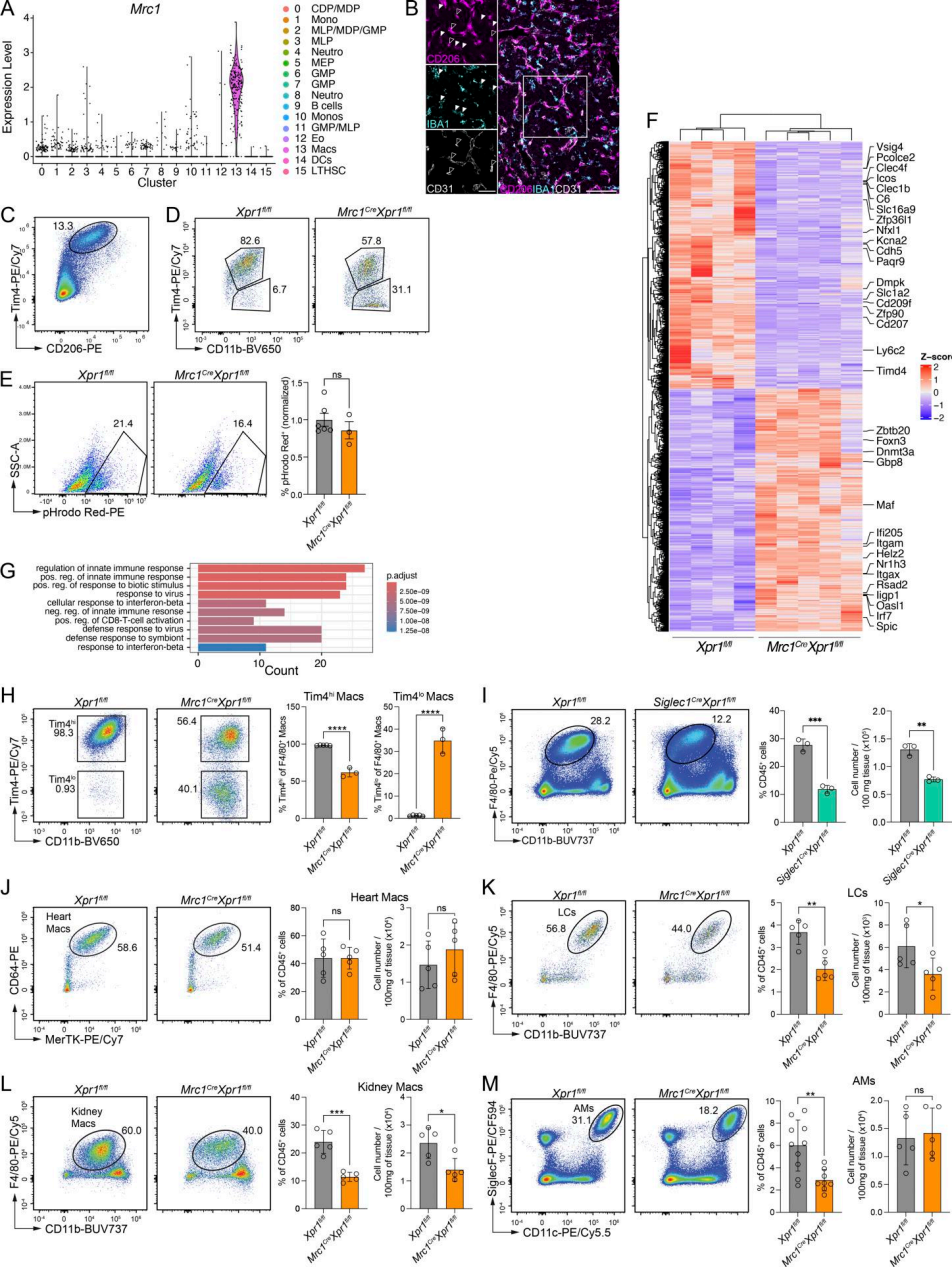
**Phenotypic and functional changes in *Mrc1*** ^***Cre***^ ***Xpr1*** ^***fl/fl***^ **animals. (A)** Violin plots showing the expression of *Mrc1* in the indicated clusters of E15.5 FL cells. Data refer to scRNA-seq data in Fig. 3 A. **(B)** Immunohistochemistry of E15.5 *Xpr1*^*fl/fl*^ control liver stained for IBA1 (cyan), CD206 (magenta), and CD31 (white). Insets: Single stainings shown of the outlined region in the overview image. Scale bar: 100 µm, inset 50 µm. Filled arrowheads show CD206^+^ macrophages, open arrowheads show CD206^+^ endothelial cells. **(C)** Representative flow cytometry plot of CD206 expression in liver macrophages extracted from E15.5 *Xpr1*^*fl/fl*^ embryos (pre-gated on CD45^+^CD31^−^Ly6G^−^F4/80^+^CD11b^+^TER119^−^ cells). **(D)** Representative flow cytometry plots of Tim4 expression in liver macrophages extracted from E15.5 *Xpr1*^*fl/fl*^ and *Mrc1*^*Cre*^*Xpr1*^*fl/fl*^ embryos (pre-gated on CD45^+^Ly6G^−^F4/80^+^ cells). Data are representative of at least *n* = 5 per group. **(E)** Representative flow cytometry plots and graph showing the percentage (normalized to control) of pHrodo Red^+^ FL macrophages (gated on CD45^+^F4/80^+^CD11b^int^ cells) from *Mrc1*^*Cre*^*Xpr1*^*fl/fl*^ and *Xpr1*^*fl/fl*^ FLs at E15.5, exposed *in vitro* to pHrodo Red Zymosan bioparticles for 90 min. Data shown are *n* = 3–6 per group and are pooled from two independent experiments. Statistical analysis was performed using the Student’s *t* test. **(F)** Heatmap of all 6,208 DEGs in the RNA-seq data of *Xpr1*^*fl/fl*^ and *Mrc1*^*Cre*^*Xpr1*^*fl/fl*^ FL macrophages from Fig. 5 E. Genes indicated are the core KC-genes and KC-associated transcription factors identified in Bonnardel et al. (2019). **(G)** Gene ontology analysis for biological processes of the DEGs in the RNA-seq data of *Xpr1*^*fl/fl*^ and *Mrc1*^*Cre*^*Xpr1*^*fl/fl*^ FL macrophages from Fig. 5 E. **(H)** Representative flow cytometry plots, frequencies, and numbers (mean ± SD) of Tim4 expression on KCs of adult *Xpr1*^*fl/fl*^ and *Mrc1*^*Cre*^*Xpr1*^*fl/fl*^ mice (pre-gated on CD45^+^Ly6G^−^F4/80^+^ cells). Data shown are *n* = 3–5 per group and are pooled from two independent experiments. Statistical analyses were performed using the Student’s *t* test. **(I)** Representative flow cytometry plots, frequencies, and numbers (mean ± SD) of KCs of adult *Xpr1*^*fl/fl*^ and *Siglec1*^*Cre*^*Xpr1*^*fl/fl*^ mice (pre-gated on CD45^+^Ly6G^−^F4/80^+^ cells). Data shown are *n* = 3 per group. Statistical analyses were performed using the Student’s *t* test. **(J–M)** Representative flow cytometry plots, frequencies, and numbers (mean ± SD) of (J) heart macrophages (Heart Macs) (pre-gated on CD45^+^Ly6G^−^ cells), (K) Langerhans cells (LCs) (pre-gated on CD45^+^Ly6G^−^TCRβ^−^SiglecF^−^Langerin^+^ cells), (L) kidney macrophages (Kidney Macs) (pre-gated on CD45^+^Ly6G^−^CD3^−^CD19^−^B220^−^CD49b^−^CD90^−^ cells), and (M) alveolar macrophages (AMs) (pre-gated on CD45^+^Ly6G^−^CD3^−^CD19^−^B220^−^CD49b^−^CD90^−^ cells) of adult *Xpr1*^*fl/fl*^ and *Mrc1*^*Cre*^*Xpr1*^*fl/fl*^ mice. Data shown are *n* = 5 per group and are pooled from two independent experiments. Statistical analyses were performed using the Student’s *t* test. *P < 0.05, **P < 0.01, ***P < 0.001, and ****P < 0.0001. ns, not significant.

**Figure 5.**
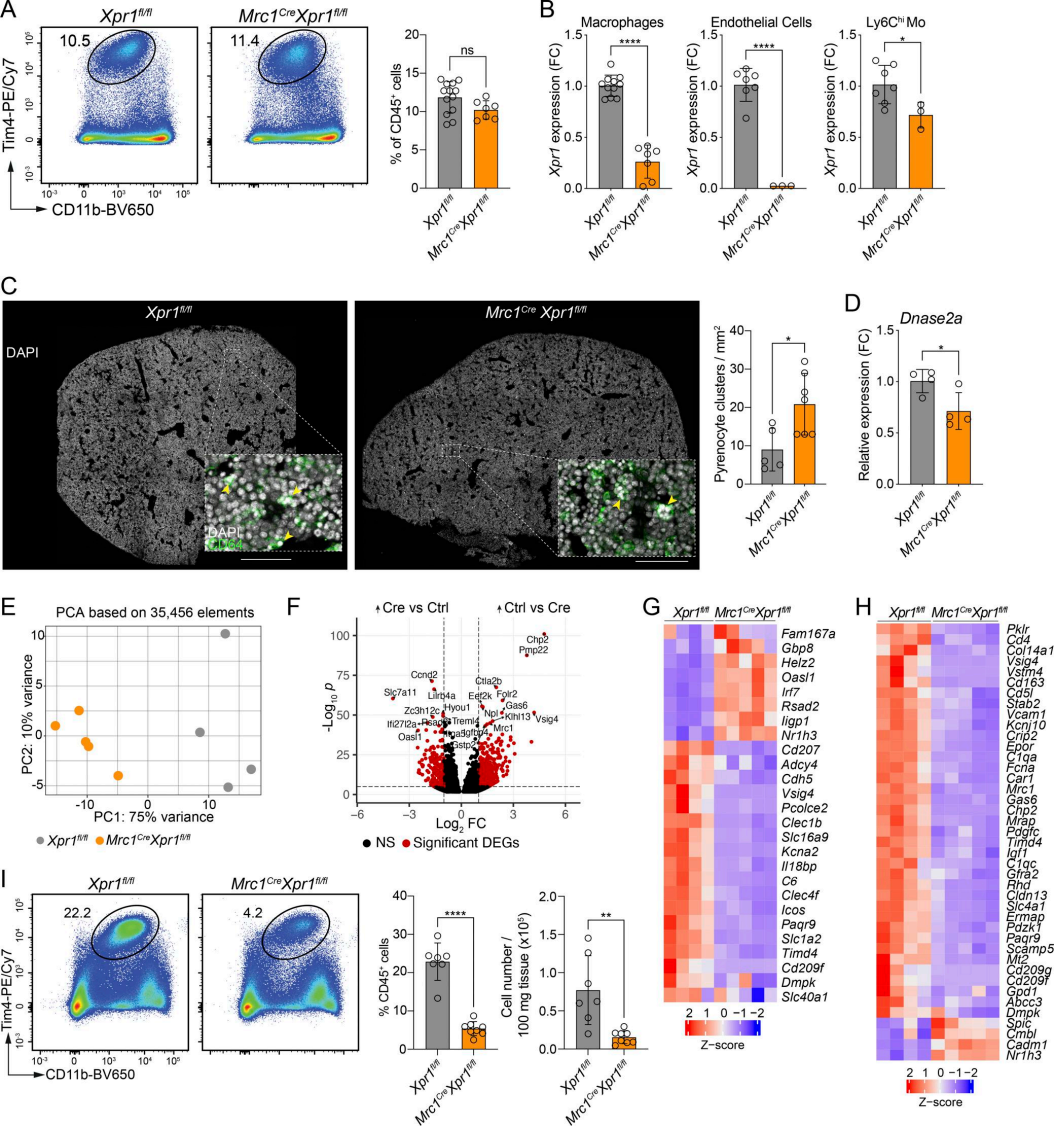
**FL macrophage function is impaired in *Mrc1*** ^***Cre***^ ***Xpr1*** ^***fl/fl***^ **embryos. (A)** Representative flow cytometry plots and frequencies (mean ± SD) of liver macrophages extracted from E15.5 *Xpr1*^*fl/fl*^ and *Mrc1*^*Cre*^*Xpr1*^*fl/fl*^ embryos (pre-gated on CD45^+^Lin^−^ cells). *n* = 11 per group, pooled from three independent experiments. Statistical analysis was performed using the Student’s *t* test. **(B)** Graphs showing *Xpr1* expression in sorted macrophages (CD45^+^F4/80^+^), endothelial cells (CD45^−^CD31^+^), and monocytes (CD11b^+^Ly6C^hi^CD64^+^) extracted from livers of E15.5 *Xpr1*^*fl/fl*^ and *Mrc1*^*Cre*^*Xpr1*^*fl/fl*^ embryos. *n* = 3–11 per group. Statistical analysis was performed using the Student’s *t* test. **(C)** Immunohistochemistry of E15.5 *Xpr1*^*fl/fl*^ and *Mrc1*^*Cre*^*Xpr1*^*fl/fl*^ livers and corresponding quantification of pyrenocyte-containing CD64^+^ cells. Insets show magnification of the outlined regions and expression of DAPI (white) and CD64 (green). Yellow arrows point to CD64^+^ cells containing pyrenocytes. Images are representative of at least five embryos per group. *n* = 5–7 per group, pooled from three independent experiments. Scale bars are 500 µm. Statistical analysis was performed using the Student’s *t* test. **(D)** Graph showing *Dnase2a* expression in sorted macrophages from livers of E15.5 *Xpr1*^*fl/fl*^ and *Mrc1*^*Cre*^*Xpr1*^*fl/fl*^ embryos. Data shown are *n* = 4 samples per group. Statistical analysis was performed using the Student’s *t* test. **(E–H)** RNA-seq data of CD11b^int^F4/80^+^ FL macrophages sorted from E15.5 *Xpr1*^*fl/fl*^ and *Mrc1*^*Cre*^*Xpr1*^*fl/fl*^ embryos. *n* = 4–5 samples per group. **(E)** PCA of *Xpr1*^*fl/fl*^ and *Mrc1*^*Cre*^*Xpr1*^*fl/fl*^ FL macrophages. Percentages shown indicate the proportion of variance explained by PC1 and PC2. **(F)** Volcano plot of DEGs between *Xpr1*^*fl/fl*^ and *Mrc1*^*Cre*^*Xpr1*^*fl/fl*^ FL macrophages. **(G)** Heatmap of core KC-gene signature from Bonnardel et al. (2019) applied to the RNA-seq data of *Xpr1*^*fl/fl*^ and *Mrc1*^*Cre*^*Xpr1*^*fl/fl*^ FL macrophages. **(H)** Heatmap of the top 50 EBI-Mac genes (Li et al., 2019) applied to the RNA-seq data of *Xpr1*^*fl/fl*^ and *Mrc1*^*Cre*^*Xpr1*^*fl/fl*^ FL macrophages. **(I)** Representative flow cytometry plots, frequencies, and numbers (mean ± SD) of liver macrophages from adult *Xpr1*^*fl/fl*^ and *Mrc1*^*Cre*^*Xpr1*^*fl/fl*^ mice (pre-gated on Live CD45^+^Lin^−^ cells). Data show *n* = 7–8 per group and are pooled from three independent experiments. Statistical analysis was performed using the Student’s *t* test. *P < 0.05, **P < 0.01, and ****P < 0.0001. ns, not significant.

To circumvent the developmental role of XPR1 in FL macrophages, we generated *Mrc1*^*Cre*^*Xpr1*^*fl/fl*^ animals, where *Xpr1* is deleted in differentiated CD206^+^ macrophages but not in EMPs or in monocytes. CD206 (encoded by *Mrc1*) is expressed by most KCs in development and is also highly expressed by liver sinusoidal endothelial cells (Guilliams et al., 2022), which is supported by our scRNA-seq data, flow cytometry, and immunohistochemistry from E15.5 embryos (Fig. S5, A–C). Analysis of embryonic livers revealed largely unchanged macrophage frequencies between *Mrc1*^*Cre*^*Xpr1*^*fl/fl*^ and *Xpr1*^*fl/fl*^ embryos (Fig. 5 A). However, a Tim4^lo^ population was present in *Mrc1*^*Cre*^*Xpr1*^fl/fl^ mice in addition to the Tim4^hi^ cells seen in control embryos (Fig. S5 D). To check gene targeting efficiency, we sorted macrophages, Ly6C^hi^ monocytes, and endothelial cells from the FL of E15.5 embryos and performed qPCR. *Xpr1* mRNA was significantly reduced in *Mrc1*^*Cre*^*Xpr1*^*fl/fl*^ compared to *Xpr1*^*fl/fl*^ macrophages (Fig. 5 B). Similarly, *Xpr1* expression was highly reduced in *Mrc1*^*Cre*^*Xpr1*^*fl/fl*^ endothelial cells and only marginally reduced in Ly6C^hi^ monocytes (Fig. 5 B). We next assessed whether *Mrc1*^*Cre*^*Xpr1*^*fl/fl*^ embryos displayed a similar impairment in pyrenocyte clearance as seen in *Vav1*^*iCre*^*Xpr1*^*fl/fl*^ embryos above. We indeed noted increased numbers of pyrenocyte clusters in *Mrc1*^*Cre*^*Xpr1*^*fl/fl*^ livers (Fig. 5 C). Although the phenotype was less pronounced than in *Vav1*^*iCre*^*Xpr1*^*fl/fl*^ embryos, the findings nevertheless support the conclusion that XPR1 is intrinsically required in macrophages for efficient clearance of expelled nuclei during erythropoiesis. This was unlikely due to impaired phagocytic capacity per se, as FL macrophages from *Mrc1*^*Cre*^*Xpr1*^*fl/fl*^ and *Xpr1*^*fl/fl*^ mice exhibited comparable uptake of pHrodo Red Zymosan bioparticles *ex vivo* (Fig. S5 E). However, similar to *Vav1*^*iCre*^*Xpr1*^*fl/fl*^ mice, *Dnase2a* expression was significantly reduced in *Mrc1*^*Cre*^*Xpr1*^*fl/fl*^ FL macrophages at E15.5 (Fig. 5 D), suggesting a defect in the degradation of pyrenocytes.

Next, to better understand how *Xpr1* deletion in FL macrophages affects their function, we sorted F4/80^+^ macrophages (including both Tim4^hi^ and Tim4^lo^ populations) from E15.5 *Xpr1*^*fl/fl*^ and *Mrc1*^*Cre*^*Xpr1*^*fl/fl*^ FLs and performed bulk RNA-seq. Principal component analysis (PCA) of the sorted cells revealed a distinct separation of the genotypes (Fig. 5 E). 6,104 genes were significantly changed in macrophages from *Mrc1*^*Cre*^*Xpr1*^*fl/fl*^ FLs when compared to *Xpr1*^*fl/fl*^ controls (Fig. 5 F and Fig. S5 F). Of these, 3,089 were significantly upregulated and 3,015 were downregulated. Among the differentially expressed genes (DEGs), we observed a profound reduction in FL/KC-identity genes including *Vsig4*, *Clec4f*, *Timd4*, and *Cdh5* in the absence of *Xpr1* (Fig. 5 G). Concurrently, IFN-stimulated genes *Oasl1*, *Irf7*, *Rsad2*, *Iigp1*, and *Helz2* were increased in *Mrc1*^*Cre*^*Xpr1*^*fl/fl*^ versus *Xpr1*^*fl/fl*^ macrophages. The source of type I IFNs in this model remains unclear, as *Ifn* gene expression was undetectable in the scRNA-seq data in the macrophages. Gene ontology analysis of genes upregulated in *Mrc1*^*Cre*^*Xpr1*^*fl/fl*^ also revealed a strong association with the biological processes of “innate immune regulation” and “interferon signaling” (Fig. S5 G), consistent with the observed pyrenocyte accumulation and our findings in *Vav1*^*iCre*^*Xpr1*^*fl/fl*^ livers.

By comparing the top 50 DEGs of EBI-Macs (Li et al., 2019), we noted that although the majority of the EBI-Mac signature was lost in the absence of *Xpr1* (Fig. 5 H), several FL macrophages/KC identity genes (such as *Spic*, *Cmbl*, *Cadm1*, and *Nr1h3*) (Sakai et al., 2019) were increased in *Mrc1*^*Cre*^*Xpr1*^*fl/fl*^ macrophages. *Spic*, for instance, is essential for the differentiation of iron-recycling macrophages (Haldar et al., 2014); however, its increased expression in *Mrc1*^*Cre*^*Xpr1*^*fl/fl*^ macrophages was insufficient to restore EBI-macrophage identity. These findings indicate that XPR1 is required to maintain the EBI-Mac transcriptional program.

In contrast to *Vav1*^*iCre*^*Xpr1*^*fl/fl*^ embryos, which die shortly after birth, *Mrc1*^*Cre*^*Xpr1*^*fl/fl*^ mice survive into adulthood. We therefore investigated the fate of liver macrophages in this model, noting significantly reduced KCs in adult *Mrc1*^*Cre*^*Xpr1*^*fl/fl*^ livers compared to control livers (Fig. 5 I). Among the remaining F4/80^+^ liver macrophages, we noted both Tim4^hi^ and Tim4^lo^ subsets in *Mrc1*^*Cre*^*Xpr1*^*fl/fl*^ mice (Fig. S5 H), which correlated with our embryonic data.

Since *Xpr1* was also deleted in endothelial cells in the *Mrc1*^*Cre*^*Xpr1*^*fl/fl*^ model (Fig. 5 B), we sought to determine whether the reduction in KCs in adult *Mrc1*^*Cre*^*Xpr1*^*fl/fl*^ animals was due to the intrinsic loss of XPR1 in macrophages rather than endothelial cells. To address this, we analyzed *Siglec1*^*Cre*^*Xpr1*^*fl/fl*^ animals, in which Siglec1 (CD169)-expressing macrophages are targeted, but endothelial cells remain unaffected. These mice survived into adulthood and exhibited a significant reduction in KCs (Fig. S5 I), similar to *Mrc1*^*Cre*^*Xpr1*^*fl/fl*^ animals. While XPR1 may have distinct roles in various cell types, including endothelial cells, these data strongly suggest a macrophage-intrinsic requirement for XPR1 in KC development and maintenance.

### XPR1 regulates the development of red pulp and BM macrophages

**Figure 6.**
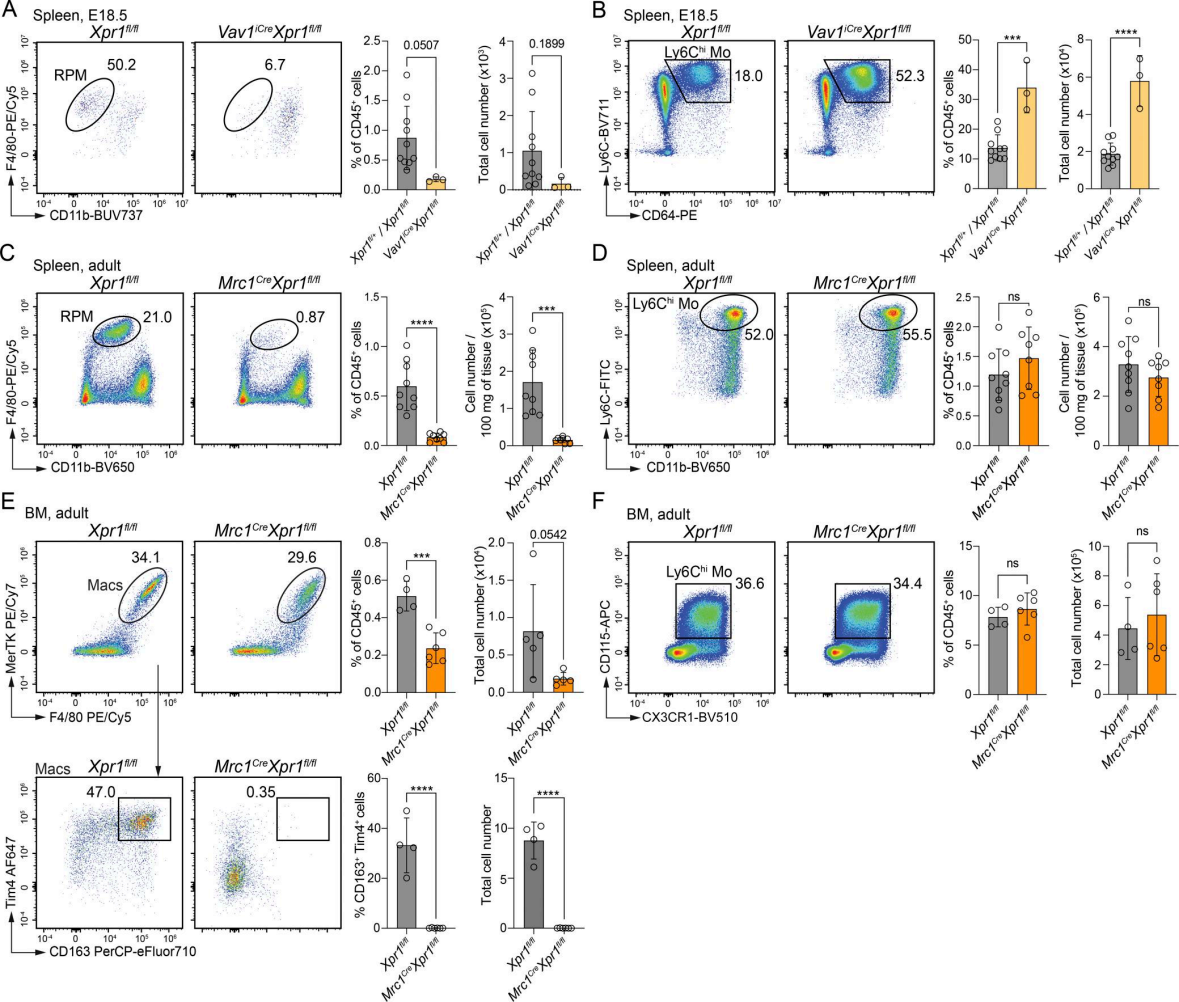
**XPR1 regulates iron-recycling macrophages in the spleen and BM. (A)** Representative flow cytometry plots, frequencies, and numbers (mean ± SD) of splenic macrophages from E18.5 control (*Xpr1*^*fl/fl*^ and *Xpr1*^*fl/+*^) and *Vav1*^*iCre*^*Xpr1*^*fl/fl*^ embryos (pre-gated on CD45^+^Lin^−^Ly6C^−^Ly6G^−^SiglecF^−^CD64^+^ cells). *n* = 3–10 per group, pooled from two independent experiments. Statistical analysis was performed using the Student’s *t* test. **(B)** Representative flow cytometry plots, frequencies, and numbers (mean ± SD) of splenic Ly6C^hi^ monocytes from E18.5 *Xpr1*^*fl/fl*^ and *Vav1*^*iCre*^*Xpr1*^*fl/fl*^ embryos (pre-gated on CD45^+^Lin^−^Mac^−^SiglecF^−^ cells). Data show *n* = 3–10 per group and are pooled from two independent experiments. Statistical analysis was performed using the Student’s *t* test. **(C)** Representative flow cytometry plots, frequencies, and numbers (mean ± SD) of splenic red pulp macrophages (RPMs) from adult *Xpr1*^*fl/fl*^ and *Mrc1*^*Cre*^*Xpr1*^*fl/fl*^ mice (pre-gated on CD45^+^Lin^−^Ly6G^−^SiglecF^−^CD90^−^MHCII^−^ cells). Data show *n* = 3–5 per group and are pooled from two independent experiments. Statistical analysis was performed using the Student’s *t* test. **(D)** Representative flow cytometry plots, frequencies, and numbers (mean ± SD) of splenic Ly6C^hi^ monocytes from adult *Xpr1*^*fl/fl*^ and *Mrc1*^*Cre*^*Xpr1*^*fl/fl*^ mice (pre-gated on CD45^+^Lin^−^Ly6G^−^SiglecF^−^CD90^−^B220^−^CX3CR1^+^CD64^+^ cells). Data show *n* = 3–5 per group and are pooled from two independent experiments. Statistical analysis was performed using the Student’s *t* test. **(E)** Representative flow cytometry plots, frequencies, and numbers (mean ± SD) of F4/80^+^MerTK^+^ BM macrophages (top) (pre-gated on CD45^+^Lin^−^Ly6C^−^Ly6G^−^) and among these, CD163^+^TIM4^+^ macrophages (bottom) from adult *Xpr1*^*fl/fl*^ and *Mrc1*^*Cre*^*Xpr1*^*fl/fl*^ mice. Data show *n* = 4–6 per group and are pooled from two independent experiments. Statistical analysis was performed using the Student’s *t* test. **(F)** Representative flow cytometry plots, frequencies, and numbers (mean ± SD) of BM monocytes (CD115^+^CX3CR1^+^) from adult *Xpr1*^*fl/fl*^ and *Mrc1*^*Cre*^*Xpr1*^*fl/fl*^ mice (pre-gated on CD45^+^Lin^−^ Ly6G^−^Ly6C^+^SiglecF^−^). Data show *n* = 4–6 per group and are pooled from two independent experiments. Statistical analysis was performed using the Student’s *t* test. ***P < 0.001 and ****P < 0.0001. ns, not significant.

Given the overlapping transcriptional signatures and functional roles of FL macrophages and RPMs (Bonnardel et al., 2019), we investigated whether RPMs were affected in the *Vav1*^*iCre*^*Xpr1*^*fl/fl*^ model. Similar to FL macrophages at E15.5, we found a marked reduction of RPMs in *Vav1*^*iCre*^*Xpr1*^*fl/fl*^ spleens at E18.5 (Fig. 6 A), and we also observed the previously identified increase in monocytes in *Vav1*^*iCre*^*Xpr1*^*fl/fl*^ E18.5 spleens compared to control embryos (Fig. 6 B). These findings suggest that XPR1 might be universally required for the development and function of all EBI-Macs, or at least macrophages with functions involved in RBC development and iron recycling.

To address this hypothesis, we analyzed spleens and BM of adult *Mrc1*^*Cre*^*Xpr1*^*fl/fl*^ mice. We observed a dramatic reduction of RPMs in adult *Mrc1*^*Cre*^*Xpr1*^*fl/fl*^ compared to control animals (Fig. 6 C). Unlike the increase of splenic monocytes in *Vav1*^*iCre*^*Xpr1*^*fl/fl*^ embryos, however, monocytes in adult *Mrc1*^*Cre*^*Xpr1*^*fl/fl*^ animals were unchanged compared to controls (Fig. 6 D). In the BM, we saw an overall reduction of F4/80^hi^MerTK^+^ macrophages (Fig. 6 E, top), among which there was a complete loss of the CD163^+^Tim4^+^ subset (Fig. 6 E, bottom). CD163 is a hemoglobin-haptoglobin scavenger receptor that is highly expressed on EBI-Macs including RPMs and KCs (Li et al., 2019). Similar to what was observed in the spleen, monocytes were not affected in the BM of *Mrc1*^*Cre*^*Xpr1*^*fl/fl*^ mice (Fig. 6 F). The maintenance of heart macrophages was not dependent on XPR1 (Fig. S5 J); however, we observed partial reductions in LCs, kidney, and alveolar macrophages in adult *Mrc1*^*Cre*^*Xpr1*^*fl/fl*^ mice (Fig. S5, K–M), suggesting that these macrophages were also sensitive to the loss of XPR1. Overall, our data show that XPR1 is a novel factor required for the development and function of EBI-Macs.

## Discussion

Here, we show a requirement for XPR1 in EBI-Mac development and function. Deletion of *Xpr1* in *Vav1*^*+*^ hematopoietic cells led to a complete loss of FL macrophages and splenic RPMs and the appearance of more undifferentiated, inflammatory Mo/Macs. Furthermore, deletion of *Xpr1* in CD206-expressing cells resulted in altered FL macrophage transcriptional identity and function, and reduced KC, RPM, and BM macrophage numbers in adult mice. The absence of FL macrophages in anti-CSF1R antibody depletion models or gene-deficient mice (*Maea*^*−/−*^, *Tnfrsf11a*^*Cre/+*^*Spi1*^*f/f*^) results in disturbed erythropoiesis (Soni et al., 2006; Kayvanjoo et al., 2024). Here, in the absence of XPR1 and/or FL macrophages, erythrocyte development appeared normal, suggesting that the alternate macrophages compensate for their absence. Indeed, the signals inducing erythroblast enucleation remain elusive but have been proposed to include soluble factors, cell-to-cell contact with macrophages, and erythroblast intrinsic factors such as Emp and Klf1 (reviewed in Moras et al. [2017]). Regardless of whether FL macrophages (or the alternate Mo/Macs) provide the enucleation signal, we demonstrate that XPR1 in FL macrophages is required for pyrenocyte clearance, which was impaired in our models. However, how *Xpr1* expression is linked to genes associated with pyrenocyte clearance, including *Dnase2*, *Ctsl*, and *Ctsd,* remains to be determined.

Id3 is a lineage-determining transcription factor for KCs, and its absence leads to partial loss of these cells (Mass et al., 2016). In *Vav1*^*iCre*^*Xpr1*^*fl/fl*^ livers, we noted the loss of an *Id3*-expressing population that is likely an FL macrophage precursor. Although *Id3* was expressed at high levels in the alternate Mo/Macs, this population failed to develop into true FL macrophages, suggesting that other factors in addition to *Id3* drive the transcriptional KC program. Similarly, Maf, a transcription factor critical for terminal macrophage differentiation (Sakai et al., 2019), and expression of EBI-Mac genes (including *Vcam1*, *Mrc1*, *Csf1r*, and *Sell*) (Kusakabe et al., 2011), was highly expressed in the alternate Mo/Macs. Yet, we found no expression of these EBI signature genes in *Maf*-expressing alternate Mo/Macs, suggesting that XPR1 is a non-redundant factor for the FL macrophage/KC-specific transcriptional program. Furthermore, the alternate Mo/Macs lacking *Xpr1* displayed a type I IFN-responsive gene signature. Whether these macrophages themselves produce type I IFNs, similar to *Dnase2*^−/−^ macrophages in the FL, which fail to degrade pyrenocyte DNA (Yoshida et al., 2004), remains to be shown.

We observed that deletion of *Xpr1* in CD206-expressing KCs resulted in a macrophage population that was heterogenous for *Tim4* in both embryos and adults. Tim4 is an apoptotic receptor for “eat-me” signals, is expressed early during FL macrophage/KC development, and is among the top KC markers. It was previously shown that, following the depletion of KCs, infiltrating monocytes quickly upregulate KC signature genes such as *Clec4f*, *Nr1h3*, *Id3*, and *Spic*; however, *Timd4*/Tim4 levels on repopulating macrophages did not reach pre-depletion levels even after 1 mo (Bonnardel et al., 2019; Sakai et al., 2019; Scott et al., 2016). This suggests that Tim4^−^ liver macrophages are monocyte-derived and/or immature KCs. Indeed, KC-specific deletion of *Nr1h3*, which encodes the nuclear cholesterol receptor and KC lineage-determining transcription factor Liver X receptor α (Sakai et al., 2019; Scott et al., 2018), led to a similar phenotype of *Tim4*^+^ and *Tim4*^−^ KCs. In contrast to our *Mrc1*^*Cre*^*Xpr1*^*fl/fl*^ model, however, deletion of *Nr1h3* in Clec4f-expressing KCs did not lead to reduced KC numbers in the liver (Sakai et al., 2019). Our data therefore indicate that XPR1 is required for the survival and homeostasis of fetal macrophages/KCs and that, in its absence, monocytes fail to fully develop into *bona fide* KCs.

The mechanism by which XPR1 promotes EBI-Mac development and programming remains unclear. XPR1 has only recently been shown to be a definitive phosphate exporter (Yan et al., 2024; Lu et al., 2024; Zhang et al., 2025; He et al., 2025). SPX-domain-containing proteins (such as eukaryotic phosphate signaling proteins and transporters including XPR1) in plants have been shown to interact with transcription factors (Wild et al., 2016). Furthermore, as high concentrations of extracellular phosphate are cytotoxic, increased accumulation of phosphate within *Xpr1*-deficient macrophages or their precursors could lead to apoptosis (Alexander et al., 2022). However, it is not clear why EBI-Macs in particular would be sensitive to dysregulated phosphate homeostasis. Whether the intracellular phosphate-sensing ability of XPR1 is required for its role in EBI-Mac development is also not resolved. We show that the absence of *Xpr1* in uncommitted precursors leads to an arrest in the differentiation to EBI-Macs, causing them to acquire an alternate Mo/Mac program. A role for XPR1 as an EBI-Mac developmental and identity factor is also supported by our finding in *Mrc1*^*Cre*^*Xpr1*^*fl/fl*^ mice, where deletion of *Xpr1* in *Mrc1*/CD206-expressing cells led to a downregulation of canonical EBI-Mac genes. In addition to its function in EBI-Macs, we demonstrate that XPR1 also plays a role in the maintenance of AMs and kidney macrophages in adulthood. In zebrafish, the development of some TRMs was also shown to be impaired in *Xpr1b* mutants (Meireles et al., 2014), demonstrating a specific role for XPR1 in the macrophage lineage across species.

Taken together, we describe that XPR1 is critical for the differentiation, fate, and function of murine EBI-Macs. These data may open new avenues for investigating the function of XPR1 in mononuclear phagocytes and its role in supporting the EBI-Mac niche in development, health, and disease.

## Materials and methods

### Mice

C57BL/6JRj mice were purchased from Janvier Labs. The *Xpr1*^*LacZ*^ (C57BL/6N-*Xpr1*^*tm1a(KOMP)Wtsi*^) mouse strain was obtained through the Knock-Out First Consortium (KOMP repository) (MGI accession no. 4362650). These mice carry a transgenic *LacZ*-Neo cassette upstream of exon 2 of *Xpr1*, flanked by FRT sites (Fig. S1 A). Exon 2 is flanked by *loxP* sites. Heterozygous *Xpr1*^*LacZ/+*^ mice carry one functional *Xpr1* allele and one non-functional *Xpr1* allele with a *LacZ* reporter, while homozygous *Xpr1*^*LacZ/LacZ*^ mice are complete knock-outs for *Xpr1*. Crossing a Xpr1^LacZ^ mouse to a Flp-recombinase mouse excises the *LacZ-Neo* cassette, resulting in a conditional *Xpr1*^*fl*^ strain (Fig. S1 A).

*Mrc1* ^*Cre*^ mice were generated by inserting a transgenic construct into Exon 30 of the *Mrc1* locus using CRISPR technology, as described previously (Van Hove et al., 2025). The targeting construct contained an internal ribosomal entry site, the codon-optimized iCre sequence, self-cleaving 2A peptide, and eYFP sequence and was synthesized by GenScript. Fertilized C57BL/6J oocytes were injected with linear-dsDNA repair targeting fragment (10 ng/μl) and Cas9 RNP (50 ng/μl). Subsequent litters were genotyped for the transgenic insertion and backcrossed to C57BL/6JRj mice for at least three generations. The whole insertion region, including surrounding genomic DNA, was sequenced to verify correct integration.

*Vav1* ^*iCre*^ (de Boer et al., 2003), *CD169*^*Cre*^ (Karasawa et al., 2015), and *Mrc1*^*Cre*^ mice were bred in-house. All strains used were backcrossed onto the C57BL/6JRj strain for at least 10 generations. All Cre strains were used as heterozygotes unless otherwise indicated. Mice were kept in individually ventilated cages under specific-pathogen-free conditions with a 12-h light-dark cycle, under controlled temperature (21–24°C) and humidity (30–70%). All experimental animal procedures at the University of Zurich were performed in accordance with the Swiss Federal regulations and approved by the Cantonal Veterinary Office of Zurich.

### Timed pregnancies

2- to 5-month-old mice were mated overnight and separated early the next morning. The morning after mating was considered E0.5.

### Cell suspension preparation

Pregnant dams were euthanized by CO_2_ inhalation and the embryo-containing uterus was removed. For blood collection, embryos were decapitated, and the blood was collected into 3 μl of 0.5 M EDTA in a 24-well plate. Embryonic organs were removed under a stereoscopic dissection microscope. Organs were finely dissociated using scissors and digested in HBSS (with Ca^2+^/Mg^2+^) containing 2% FCS, 0.4 mg/ml Collagenase IV (Worthington), and 0.04 mg/ml DNAse I (Sigma-Aldrich) for 30 min in a shaking incubator at 37°C. Samples were washed in cold PBS and erythrocytes were lysed in RBC-lysis solution (155 mM NH_4_Cl, 11 mM KHCO_3_). Samples were washed with PBS and subsequently ready for flow cytometric staining.

Adult mice were euthanized by CO_2_ inhalation and perfused with cold PBS. Mouse livers were dissociated with scissors and digested in HBSS (with Ca^2+^/Mg^2+^) containing 2% FCS, 1 mg/ml Collagenase A (Sigma-Aldrich), and 0.04 mg/ml DNAse I for 30 min in a shaking incubator at 37°C. Mouse spleens were dissociated with scissors and digested in HBSS (with Ca^2+^/Mg^2+^) containing 2% FCS, 0.4 mg/ml Collagenase IV, and 0.04 mg/ml DNAse I for 30 min in a shaking incubator at 37°C. Samples were washed in cold PBS and erythrocytes were lysed in RBC-lysis solution. Samples were washed with PBS and were subsequently ready for flow cytometric analysis.

### FL-derived macrophage generation

E14.5 FLs were homogenized with a P1000 pipette and 2.5 × 10^6^ cells seeded into each well of a 6-well plate in DMEM supplemented with 10% FCS (Thermo Fisher Scientific), 100 U/ml pen/strep (Thermo Fisher Scientific), and 20 ng/ml CSF-1 (Peprotech). Cells were incubated at 37°C and 5% CO_2_. On day 4 after seeding, an equal volume of fresh media supplemented with CSF-1 was added to each well. On day 7 after seeding, cells were washed in PBS, scraped off the plate and analyzed by flow cytometry.

### Flow cytometry

Cells were incubated with anti-mouse CD16/32 (clone 93, 101310; BioLegend) and Zombie NIR Live/Dead fixable viability stain (BioLegend) for 20 min at 4°C in the dark to block the Fc-receptor and stain dead cells, respectively. Cells were then stained with fluorochrome-conjugated antibodies in FACS Wash (PBS containing 2% FCS and 2 mM EDTA) for 30 min at 4°C in the dark. Antibodies used were I-A/I-E (clone M5/114.15.2; PacificBlue, 107620; BioLegend; BV605, 107639; BioLegend; BV510, 107636; BioLegend), CD31 (clone 390, BUV805, 741949; BD; clone MEC13.3; PE, 102507; BioLegend), CD11b (clone M1/70, BUV737, 612800; BD), CD11c (clone N418, PE/Cy5.5, 35-0114-82; eBioscience; PE/Cy7, 117318; BioLegend; BV570, 117331; BioLegend; APC, 117310; BioLegend), CD45 (clone 30-F11, BUV395, 564279; BD; AF700, 103128; BioLegend), Ly6G (clone 1A8, BV650, 127641; BioLegend; BV785, 127645; BioLegend; V450, 560603; BD; BUV563, 565707; BD), Ly6C (clone HK1.4, BV711, 128037; BioLegend; APC, 128016; BioLegend; clone AL-21, FITC, 553104; BD), Sca1 (clone D7, BV510, 108129; BioLegend), CD48 (clone HM48-1, AF700, 103426; BioLegend), CD34 (clone SA376A4, BV421, 152208; BioLegend), CD115 (clone AFS98, PE/Cy7, 25-1152-82; eBioscience; APC, 17-1152-82; eBioscience), CD88 (clone 20/70, BV750, 747227; BD), CD90.2 (clone 30-H12, APC, 140312; BioLegend), CD49b (clone DX5, APC, 108910; BioLegend), CD62L (clone MEL-14, BUV737, 612833; BD; BV570, 104433; BioLegend), CD117 (clone 2B8, PE-EFluor610, 61-1171-82; eBioscience), CD3 (clone 17A2, APC, 100236; Biolegend), NK1.1 (clone PK136, BV711, 108745; BioLegend), B220 (clone RA3-6B2, APC, 103212; BioLegend), CD19 (eBio1D3, APC, 17-0193-82; eBioscience), CD150 (clone TC15-12F12.2, BV785, 115937; BioLegend), SiglecF (clone E50-2440, PE/CF594, 562757; BD), CD64 (clone X54-5/7.1, PE, 139304; BioLegend; BV421, 139309; BioLegend; BV711, 139311; BioLegend), F4/80 (clone BM8, PE/Cy5, 123112; BioLegend; BV510, 123135; BioLegend), CD169 (clone SER-4, BV605, 142413; Biolegend; AF488, 53-5755-82; eBioscience), CD163 (clone TNKUPJ, PerCP-EFluor710, 46-1631-82; eBioscience; PE, 12-1631-82; eBioscience), CD206 (clone C068C2, AF700, 141734; BioLegend; PE, 141706; BioLegend), Lyve1 (clone ALY7, eF450, 48-0443-82; eBioscience; AF488, 53-0443-80; eBioscience), CD38 (clone 90, PE/Dazzle594, 102729; BioLegend), CX3CR1 (clone SA011F11, BV605, 149027; BioLegend; BV510, 149025; BioLegend; PE/Dazzle594, 149013; BioLegend, Gr-1 (clone RB6-8C5, PE, 12-5931-83; eBioscience; BUV805, 741920; BD), MerTK (clone DS5MMER, SB780, 78-5751-82; eBioscience; PE/Cy7, 25-5751-82; eBioscience), Tim4 (clone F31-5G3, PE/Cy7, 130009; BioLegend; AF647, 130007; BioLegend; BV805, 749133; BD; BUV395, 745685; BD), Ter119 (clone Ter119, APC, 17-5921-81; eBioscience; BV650, 116235; BioLegend), VCAM1 (clone 429, FITC, 553332; BD), CD71 (clone RI7217, APC, 113819; BioLegend; clone C2, RB780, 755614; BD), and EPCAM (clone G8.8, PE/Cy7, 118216; BioLegend).

All flow cytometry data were acquired on a BD LSRII Fortessa, BD FACSymphony, or Cytek Aurora and analyzed with FlowJo v10 software (Tree Star). Flow cytometric cell sorting was performed on either a BD FACSAria III or a BD FACSymphony S6 using a 100 µm nozzle. Cells were sorted into 100% FCS before adjusting the volume with PBS. Sorted cells were pelleted by centrifugation, the supernatant aspirated, and the cells resuspended in RNA lysis buffer (Zymo Research).

### Flow cytometry high-dimensional analysis

Dead cells and doublets were excluded from all downstream analyses. Raw flow cytometry data were compensated using FlowJo, followed by transformation and normalization in R. Dimensionality reduction was performed using the UMAP algorithm. The FlowSOM clustering algorithm was used for population clustering. Cluster frequencies and heatmaps were generated in R.

### Blood analysis

Peripheral blood was analyzed on a Scil Vet ABC Plus blood counter. Blood smears were made by streaking 6 μl of blood along a glass slide using a coverslip. Blood smears were imaged on a Zeiss AxioScan Z1.

### Phagocytosis assay

FL cells were isolated and stained for flow cytometry as described above. Following staining, cells were resuspended in RPMI with 10% FBS and incubated with pHrodo Red Zymosan BioParticles (P35364; Invitrogen) for 1.5 h at 37°C and 5% CO_2_. After the incubation, cells were immediately analyzed by flow cytometry.

### Histology

Livers from mouse embryos were fixed with 4% paraformaldehyde for 6 h at room temperature. Livers were rinsed in PBS, then cryoprotected in 30% sucrose/PBS for 48 h at 4°C. Samples were embedded in OCT (Sakura). Sections were cut at 10 or 20 µm thickness using a Hyrax C60 cryostat (Zeiss) and transferred onto Superfrost Plus slides (Thermo Fisher Scientific). For staining, sections were blocked and permeabilized by incubation in staining buffer (1% normal donkey serum or 3% normal goat serum, 0.5% Triton X-100, and 0.5% BSA in PBS) for 30 min at room temperature. Slides were incubated with primary antibodies overnight at 4°C in a staining buffer (3% normal goat serum in PBS). Primary antibodies used were F4/80 (clone BM8, 123101; BioLegend; clone CI:A3-1, MCA497A488; Bio-Rad), CD64 (clone AT152-9, MCA5997; Bio-Rad), IBA1 (ABIN2857032; Antibodies Online), TIM4 (AF2826; R&D Systems), CD31 (AF3628; R&D Systems), and CD206 (GTX42265; GeneTex). Samples were washed three times in PBS. For secondary antibody staining, slides were incubated for 2 h at room temperature with secondary antibody diluted in staining buffer. Secondary antibodies used were goat anti-rat AF488 (A11006; Thermo Fisher Scientific) and goat anti-rat AF647 (A21247; Thermo Fisher Scientific), donkey anti-rabbit AF488 (A32790; Thermo Fisher Scientific), donkey anti-goat AF555 (A21432; Thermo Fisher Scientific), and donkey anti-rat AF647 (A78947; Thermo Fisher Scientific). Slides were washed three times and sections mounted with DAPI-containing ImmunoSelect mounting medium (Dianova) or stained for 30 min at room temperature with Hoechst 33342 (1:5,000, H3570; Thermo Fisher Scientific before mounting with ImmunoSelect mounting medium (Dianova). Images were acquired on a Leica Stellaris 5 (objective: HC PL APO CS2, 20× magnification, 0.75 numerical aperture; detectors: Power HyD S; acquisition software: Leica LSX) or Zeiss LSM980 (objective: Plan-Apochromat, 10× magnification, 0.45 numerical aperture; camera: Zeiss Axiocam 506 mono; acquisition software: ZEN 3.3 Blue Edition) or Evident FV4000 (objective: UCPLFLN 20×, 0.7 numerical aperture or UPlanXApo 40×, 0.95 numerical aperture; acquisition software: cellSENS). All images were acquired at room temperature. Images were processed using FIJI v2.9 software.

TUNEL staining was performed using the ApopTag Red In Situ Apoptosis Detection Kit (S7165; Sigma-Aldrich) according to the manufacturer’s instructions for fluorescent staining of tissue cryosections. For co-staining with antibodies, the TUNEL staining was performed first, followed by the antibody staining as previously described. Images were acquired on an Evident Fluoview 4000 microscope and TUNEL mean intensity within the CD64^+^ area was quantified using QuPath (version 0.5.1) imaging analysis software.

### scRNA-seq

E15.5 embryos were extracted from pregnant dams and FL cell suspensions prepared as described above. Embryos were genotyped and three *Xpr1*^*fl/fl*^ and three *Vav1*^*iCre*^*Xpr1*^*fl/fl*^ samples pooled. CD45^+^Lin^-^(CD19^−^CD3^−^B220^−^Ter119^−^CD49b^−^CD90.2^−^) cells were sorted as described above. The quality of the single-cell suspensions was assessed using a hemocytometer under a Leica DM IL LED Fluo microscope, and the quantity was determined using an automated cell counter (LUNA-FX7; Logos). Approximately 17,000 cells per sample were loaded onto the 10x Chromium X platform. Library preparation followed the manufacturer’s guidelines specified in the Chromium Single-Cell 3′ Reagent Kits User Guide (v3.1 Chemistry Dual Index). For sequencing, the resulting libraries were processed on an Illumina NovaSeq 6000 SP Flow Cell. Sequencing parameters were set according to 10x Genomics recommendations, using paired-end reads with the following specifications: R1 = 28, i7 = 10, i5 = 10, R2 = 90. An average depth of around 50,000 reads per cell was achieved during sequencing.

Read alignment, cell-calling, and feature-barcode count matrix generation were performed using the 10x Genomics Cell Ranger v7.1.0 pipeline. Downstream analysis was performed on the feature-barcode count matrices using the R package Seurat v4.3.0. Cells were further filtered according to UMI count, feature count, and mitochondrial percentage using a median-absolute-deviation approach implemented in the scater package. Cells with >0.7 riboprotein content and those marked as doublets by the scDblFinder package were removed. Filtered data were log-normalized and scaled using sctransform implemented in Seurat, using the top 3,000 highly variable genes. Dimensional reduction was performed using PCA. The Louvain algorithm was applied with a resolution of 0.6 to cluster the cells using the first 15 PCs. The clustered cells were visualized in a two-dimensional space via UMAP of the same PCs.

Subclustered macrophages, monocytes, and precursors were normalized, scaled, and dimension-reduced by PCA. UMAP dimensionality reduction and clustering were performed with the first 30 PCs at a resolution of 0.5. Cluster-specific genes were identified with the FindAllMarkers function and clusters annotated manually using the ImmGen database and SingleR package. FL scRNA-seq data were downloaded from Wang et al. (2020).

### Bulk RNA-seq

Between 85,000 and 100,000 macrophages (CD45^+^F4/80^+^CD11b^lo^) from *Xpr1*^*fl/fl*^ and *Mrc1*^*Cre*^*Xpr1*^*fl/fl*^ E15.5 embryos were sorted (as described above) and total RNA extracted using RNeasy Micro RNA isolation kits (QIAGEN). Sample RNA quality was analyzed on an Agilent TapeStation, and samples with an RNA integrity number value over 7.2 were used for sequencing (median sample RNA integrity number was 8.3). cDNA libraries were prepared using 45 ng of total RNA. Samples were subjected to a paired-end sequencing run of 150 reads on an Illumina NovaSeq X Plus system.

Sample reads were quality control tested using FastQC. Read count matrices were generated with the salmon package using mouse reference genome GRCm39 to build the index. Transcript reads were annotated using AnnotationHub, and data were imported using the tximport package. RNA-seq analysis was performed with DESeq2. DEGs between control and Cre^+^ samples were identified using a false discovery rate of 0.05. Gene ontology pathway analysis was performed using the clusterProfiler package.

### qRT-PCR

Lysed RNA from sorted cells was purified using the Quick-RNA Microprep Kit (Zymo Research) and reverse-transcribed using the Tetro cDNA Synthesis Kit (Meridian Bioscience). Quantitative real-time PCR was performed using SYBR Green mixes (Thermo Fisher Scientific) on a CFX384 thermal cycler (Bio-Rad). The Δ-Δ CT method was used to quantify gene expression. Primers used were 18S-F: 5′-GTA​ACC​CGT​TGA​ACC​CCA​TT-3′, 18S-R: 5′-CCA​TCC​AAT​CGG​TAG​TAG​CG-3′, Xpr1-F: 5′-CAG​GAC​CAG​GCA​CCT​TCA​GTT​G-3′, Xpr1-R: 5′-AAG​TGT​AGC​AAA​CCT​GCG​CTG​A-3′, Dnase2a-F: 5′-AAG​CCC​TGA​GCT​GCT​ATG​G-3′, and Dnase2a-R: 5′-ATA​CGT​CAG​TCC​CTT​TGG​AGT​A-3′.

### Statistical analyses

Statistical analyses were calculated using GraphPad Prism v10 software. The statistical testing methodologies are described in the figure legends. *P < 0.05, **P < 0.01, ***P < 0.001, and ****P < 0.0001; ns = not significant.

### Online supplemental material

Fig. S1 shows the cell frequencies and numbers of various monocyte and macrophage populations in *Xpr1* mutant embryos during gestation. Fig. S2 shows the expression of *Xpr1* among various liver cell populations, additional characterization of cell populations in *Vav1*^*iCre*^*Xpr1*^*fl/fl*^ embryos and immunofluorescent images of apoptotic cells in *Vav1*^*iCre*^*Xpr1*^*fl/fl*^ FLs. Fig. S3 shows extended data of the scRNA-seq analysis of *Xpr1*^*fl/fl*^ and *Vav1*^*iCre*^*Xpr1*^*fl/fl*^ FL cells, as well as additional flow cytometry data of eosinophils and alternate Mo/Macs in *Vav1*^*iCre*^*Xpr1*^*fl/fl*^ FLs. Fig. S4 depicts the characterization of FL macrophages and blood parameters in embryos depleted of macrophages using anti-CSF1R. Fig. S5 shows extended data of the RNA-seq analysis of *Xpr1*^*fl/fl*^ and *Mrc1*^*Cre*^*Xpr1*^*fl/fl*^ liver macrophages, immunofluorescent imaging data showing the co-expression of CD206, CD31, and IBA1 in FLs and additional flow cytometry data characterizing macrophage populations in *Mrc1*^*Cre*^*Xpr1*^*fl/fl*^ and *Siglec1*^*Cre*^*Xpr1*^*fl/fl*^ animals.

## Data Availability

The scRNA-seq data of E15.5 *Xpr1*^*fl/fl*^ and *Vav1*^*iCre*^*Xpr1*^*fl/fl*^ FLs are publicly available under GEO accession number GSE269382. The bulk RNA-seq data of E15.5 *Xpr1*^*fl/fl*^ and *Mrc1*^*Cre*^*Xpr1*^*fl/fl*^ macrophages are publicly available under the GEO accession number GSE269322. Further information and requests for resources and reagents should be directed to and will be fulfilled by the lead contact, Melanie Greter (greter@immunology.uzh.ch).
